# Synthesis and Biological Activity of a Cytostatic Inhibitor of MLLr Leukemia Targeting the DOT1L Protein

**DOI:** 10.3390/molecules26175300

**Published:** 2021-08-31

**Authors:** Corentin Bon, Yang Si, Melanie Pernak, Magdalena Barbachowska, Eva Levi-Acobas, Veronique Cadet Daniel, Corinne Jallet, Dusan Ruzic, Nemanja Djokovic, Teodora Djikić, Katarina Nikolic, Ludovic Halby, Paola B. Arimondo

**Affiliations:** 1 Epigenetic Chemical Biology, Department of Structural Biology and Chemistry, Institut Pasteur, UMR3523 CNRS, 75015 Paris, France; corentin.bon@pasteur.fr (C.B.); sophia.siyang@gmail.com (Y.S.); m.pernak@sfr.fr (M.P.); magdalena.barbachowska@pasteur.fr (M.B.); eva.leviacobas@gmail.com (E.L.-A.); veronique.cadet-daniel@pasteur.fr (V.C.D.); corinne.jallet@pasteur.fr (C.J.); ludovic.halby@pasteur.fr (L.H.); 2 Ecole Doctorale MTCI, Université de Paris, Sorbonne Paris Cité, 75006 Paris, France; 3 Department of Pharmaceutical Chemistry, Faculty of Pharmacy, University of Belgrade, Vojvode Stepe 450, 11000 Belgrade, Serbia; dusan.ruzic@pharmacy.bg.ac.rs (D.R.); nemanja.djokovic@pharmacy.bg.ac.rs (N.D.); teodora.djikic@pharmacy.bg.ac.rs (T.D.); katarina.nikolic@pharmacy.bg.ac.rs (K.N.)

**Keywords:** MLL rearranged leukemia, DOT1L, histone methylation, rational drug design, HMT inhibitors, bisubstrates

## Abstract

Histone methyltransferase DOT1L catalyzes mono-, di- and trimethylation of histone 3 at lysine residue 79 (H3K79) and hypermethylation of H3K79 has been linked to the development of acute leukemias characterized by the MLL (mixed-lineage leukemia) rearrangements (MLLr cells). The inhibition of H3K79 methylation inhibits MLLr cells proliferation, and an inhibitor specific for DOT1L, pinometostat, was in clinical trials (Phase Ib/II). However, the compound showed poor pharmacological properties. Thus, there is a need to find new potent inhibitors of DOT1L for the treatment of rearranged leukemias. Here we present the design, synthesis, and biological evaluation of a small molecule that inhibits in the nM level the enzymatic activity of hDOT1L, H3K79 methylation in MLLr cells with comparable potency to pinometostat, associated with improved metabolic stability and a characteristic cytostatic effect.

## 1. Introduction

Post-translational modifications of histones are involved in the epigenetic regulation and participate in the control of gene expression without altering the DNA sequence [1]. Epigenetic chemical modifications constitute a dynamic system and play a major role in normal cell development and differentiation. These epigenetic modifications have been found to be altered in several diseases such as cancer [2]. Among these modifications, the dynamic methylation of nucleosomal histones represents a complex code that will open or compact chromatin depending on the number of methyl groups (mono, di or trimethylation, symmetric or asymmetric methylation), the context (other marks on the histones and DNA) and the localization on the histones [3].

The two major families of histone methyltransferases are lysine methyltransferases (KMTs) and protein arginine methyltransferases (PRMTs) [4]. Lysine methylation is the most studied, it includes mono-, di-, and tri-methylation and the best-characterized substrates are lysine 4 on histone 3 (H3K4), lysine 9 (H3K9), lysine 27 (H3K27), lysine 36 (H3K36), and lysine 79 (H3K79), and lysine 20 on histone 4 (H4K20). Lysine methylation contributes to transcription regulation, frequently acting as landing platforms for effector proteins recruitment [5]. These modifications are often altered in cancer [6].

DOT1 (disruptor of telomeric silencing 1) and DOT1L (DOT1-like) catalyze the mono-, di-, and tri-methylation of lysine 79 of histone H3 in a non-processive manner [7]. The aberrant transcriptional activation by methylation of H3K79 by DOT1L induces transcriptional activation and it is found aberrant in Mixed-Lineage Leukemias (MLL) rearranged leukemias (MLLr). These leukemias present chromosomal rearrangements at chromosome 9 between the gene coding for the KMT MLL and different partners [8] (AF4, AF9, ENL, AF10 [9]). These rearrangements lead to the loss of the methylation activity of MLL and the recruitment of DOT1L by the partners inducing the H3K79 methylation by DOT1L at the genes targeted by MLL. The MLL gene found on chromosome 11q23 encodes a large multidomain protein complex [10], involved in the regulation of leukemia-associated genes including genes from HOX family (mainly HOXA7 [11] HOXA10 [12]) and the HOX cofactor MEIS1. The overexpression of HOXA9 and MEIS1 genes induced by the aberrant H3K79 methylation by DOT1L has been found in patients diagnosed with acute leukemias [13]. Therefore, DOT1L histone methyltransferase is a potential target in the treatment of leukemia patients. The ultimate goal is to inhibit H3K79 methylation and prevent MLLr proliferation [14]. Inhibition of DOT1L activity or disruption of DOT1L interaction with MLL fusion partners are potential therapeutic strategies for the treatment of rearranged MLL leukemia. A first compound, EPZ-4777 [15] was identified by Epizyme as a molecular inhibitor of DOT1L, competing with the SAM cofactor. This compound inhibits H3K79 methylation in cancer cells, blocks the expression of leukemogenic genes, and selectively kills cells that contain the translocation. Nevertheless, the compound has poor pharmacological properties [16]. A second-generation inhibitor, EPZ-5676 [17], entered phase I/II clinical trials for the treatment of MLL-rearranged leukemias. Metabolic instability, the low response of patients, along with some resistance led to the withdrawal of the compound in phase II [18]. Recently, compounds with novel binding modes [19,20,21] have been reported and studied in in vivo studies [22]. However, as only the substrate sugar-based analogs succeeded to go to the clinical phase, we focused on the modulation of these compounds.

**Figure 1:**
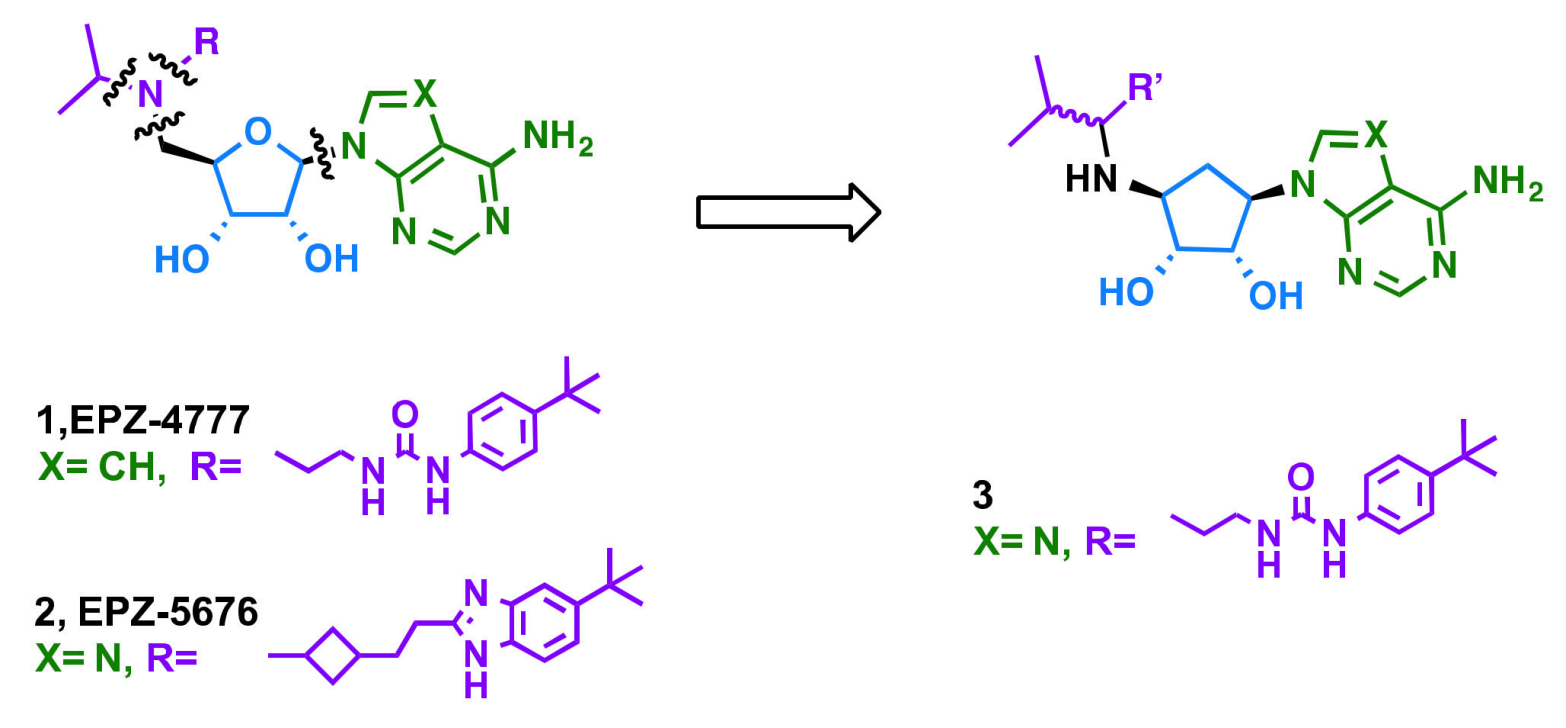
Chemical strategy: the metabolic sensitive points were replaced by more stable features. In green the adenine scaffold, in blue the sugar, and in purple the side chain specific of DOT1L inhibition.

We report here the synthesis and biological evaluation of compound **3 Dia2**, an analog of EPZ-4777, designed to improve its metabolic properties. With the aim to decrease the metabolic activity on the tertiary amine group of the C5′ position and to remove the sensitive anomeric carbon on the C2′ position [23], the ribose moiety was replaced by a 3-aminocyclopentan-1,2-diol group (Figure 1). The linker between the adenosine analog and the ter-butylaniline group was modified and coupled to the amine group of the 3-aminocyclopentan-1,2-diol moiety to conserve the same linker length. The modification induced the insertion of a chiral carbon and provided two diastereoisomers that were isolated and evaluated separately. One of them, **3 Dia2**, showed strong activity against DOT1L enzymatic activity, whereas the other had poorer activity. Molecular docking of the two diastereoisomers in the crystal structure of DOT1L suggested that the difference in their biological activity may originate from different accommodations of the isopropyl group inside the catalytic pocket. In addition, **3 Dia2**, an nM inhibitor of DOT1L, decreased H3K79 dimethylation in MLL-r cells and induced a cytostatic effect.

## 2. Results

### 2.1. Rational Design

Metabolic instability was highlighted as a major reason for EPZ-5676 (**2**) withdrawal in phase II. 5′ position *N*-dealkylation and depurination along with alkyl oxidation of the second substrate (Figure 1) were described as the main metabolic pathways for EPZ-5676 [23,24]. As alkyl hydroxylation, especially on the Tert-butyl group, has been shown to have a low effect on the potency of EPZ-5676, we focused on the sugar moiety (in blue) and the linkage of the second substrate (purple). Adenosinylcyclopentandiol was selected to avoid depurination. The tertiary amine moiety on the C5′ position was replaced by a carbon atom and a direct coupling to a hindered secondary amine on the C5 position of the cyclopentane moiety in order to lower *N*-dealkylases recognition.

### 2.2. Chemistry

**Scheme 1:**
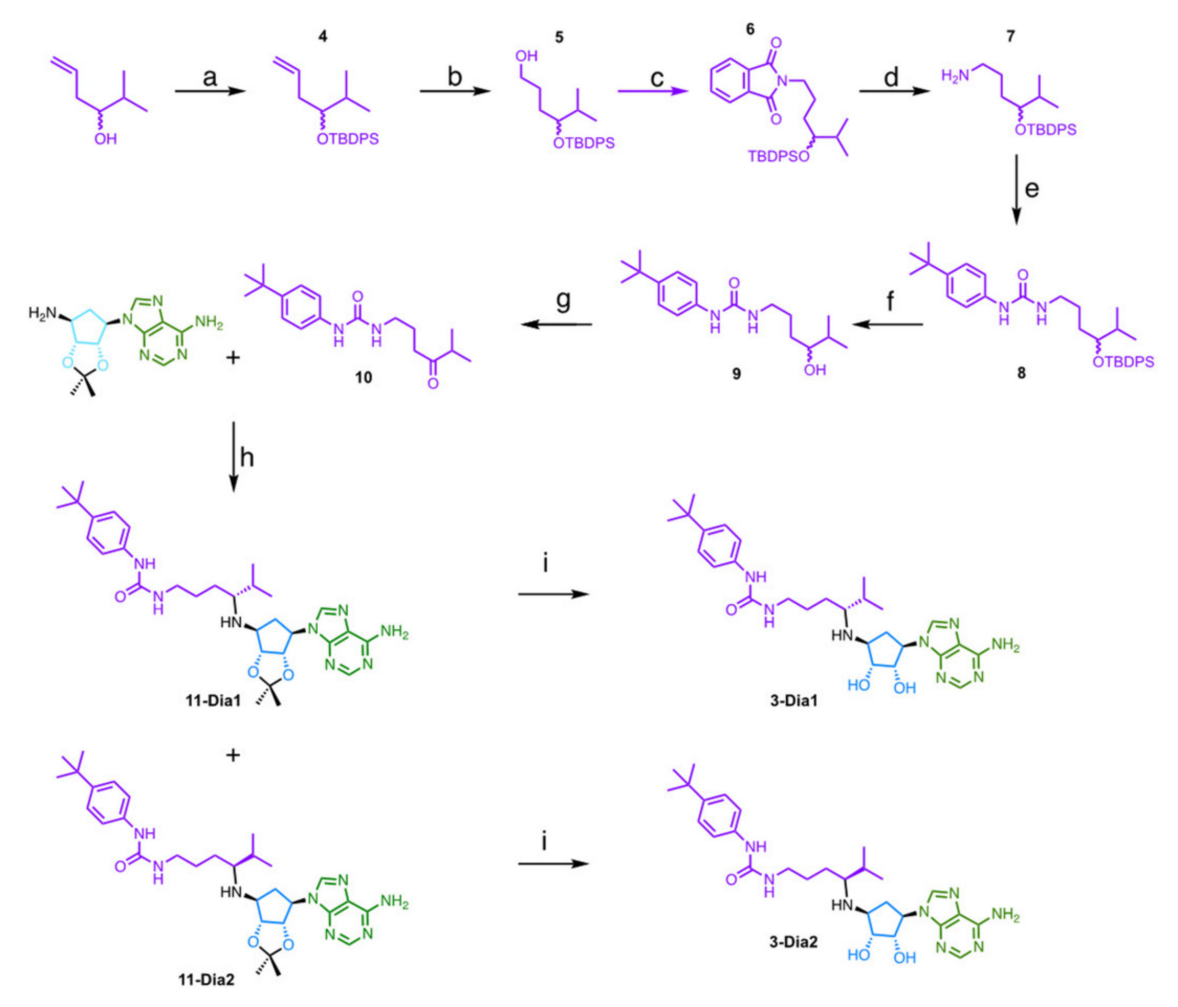
Synthesis of compounds **3**. (a) TBDPSCl, imidazole, DMF, RT, 12 h, 99%. (b) BH_3_-DMS, THF, RT, 4 h then H_2_O_2_, NaOH, water/THF, 0 °C, 12 h, 71%. (c) Phthalimide, PPh_3_, DIAD, THF, RT, 12 h, 93%. (d) MeNHNH_2_ 10% MeOH, RT, 12 h, 81%. (e) DCM, RT, 1 h, 65%. (f) TBAF, THF, O/N, 89%. (g) PCC, DCM, RT, 2 h, 71%. (h) NEt_3_, MeOH, RT, 2 h then AcOH, NaBH_3_CN, RT, 36 h, 61%. (i) TFA, H_2_O, RT, 1 h, 97%.

The target molecules **3 Dia1** and **3 Dia2** were obtained through the coupling of compound **9** with previously described (1*R*,2*S*,3*R*,4*S*)-6-amino-9-[(4-amino-20, 3-*O*-isopropylidene)cyclopent-1 yl]-9H-purine [25] (Scheme 1). The ketone derivative **9** was obtained from the commercially available 2-methyl-5-hexen-3-ol. First, the alcohol function was protected by reaction with TBDPSCl in the presence of imidazole to give compound **4**. Then, the alkene function was converted to the alcohol through a one-pot hydroboration. The resulting compound **5** was converted to the phthalimide derivative **6** by a Mitsunobu reaction with the phthalimide in the presence of DIAD and triphenylphosphine. The corresponding amine **7** was obtained by treatment of **6** with methylhydrazine. The urea formation was carried out in a one-pot reaction between **7** and the previously prepared 4-nitrophenyl-N-4-tertbutylaniline carbamate to afford compound **8**. Compound **8** was deprotected by treatment with TBAF to give the alcohol derivative **9** that was then oxidized using PCC. The ketone derivative **10** was obtained with an overall yield of 22% over 7 steps. It was then coupled by reductive amination with the (1*R*,2*S*,3*R*,4*S*)-6-amino-9-[(4-amino-20,3-*O*-isopropylidene)cyclopent-1-yl]-9H-purine leading to separable diastereomers **11 Dia1** and **11 Dia2** that upon TFA treatment afforded target compounds **3 Dia1** and **3 Dia2**, herein referred to as **Dia1** and **Dia2**. The configuration was determined by NOESY NMR spectra.

### 2.3. DOTL1 Inhibition

**Figure 2:**
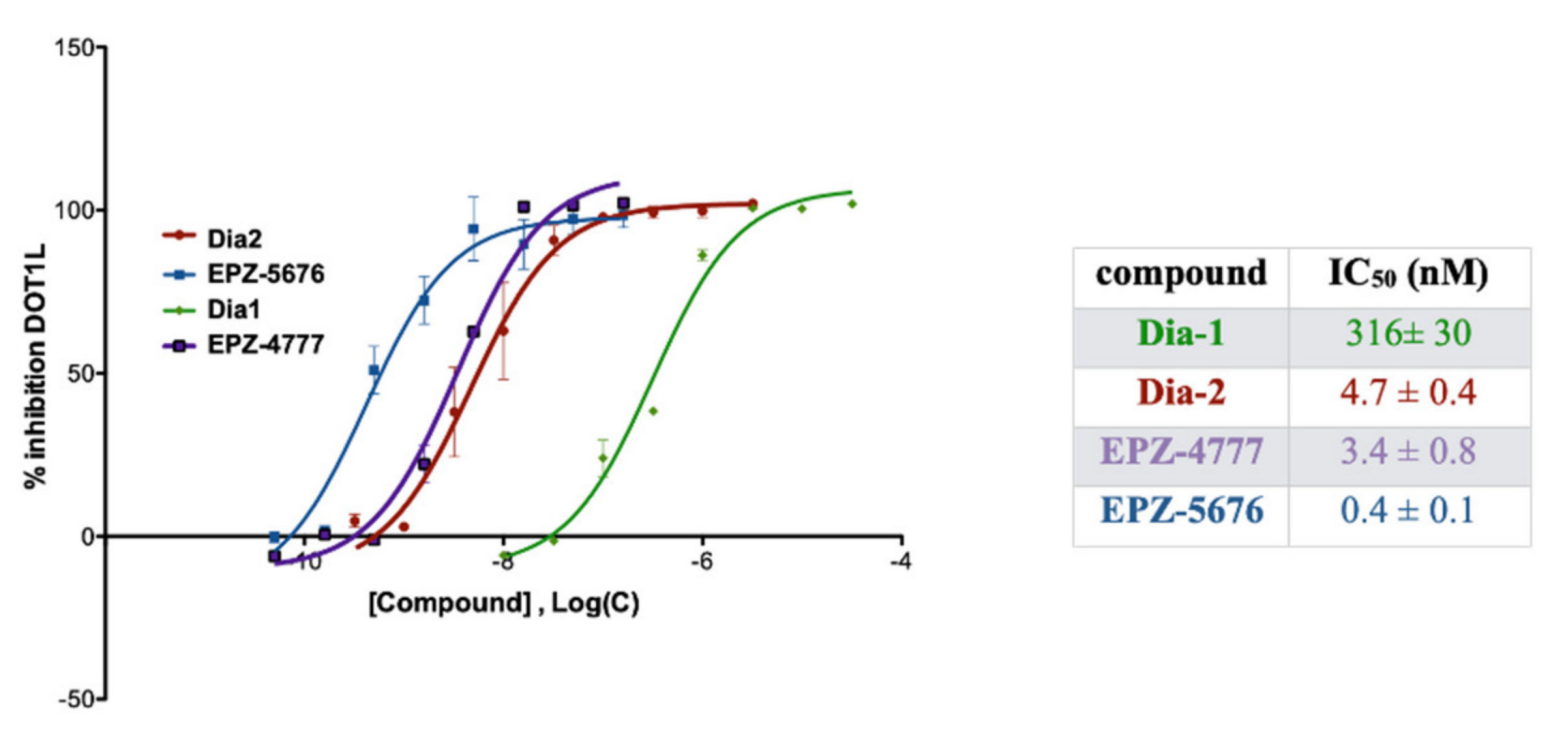
Inhibition of recombinant human DOT1L by **Dia1** (red), **Dia2** (green), EPZ-5676 (blue) and EPZ-4777 (violet). Each data point represents the mean of three technical replicates and two separated experiments at each specified concentration of the compound. The values of IC_50_ are given in nM.

**Dia1** and **Dia2** were tested in an AlphaLISA assay using recombinant hDOT1L on purified nucleosomes. EPZ-4777 **1** and EPZ-5676 **2** were used as controls and showed comparable inhibitory values as in the literature), with IC_50_ of 3.4 nM and 0.4 nM, respectively (Figure 2).

**Dia2** showed an IC_50_ of 4.7 nM in the same range as the parent compound EPZ-4777 **1**, while **Dia1** showed a drop of 67-fold in inhibitory activity with an IC_50_ of 316 nM, highlighting the importance of the conformation of the chiral carbon within the linker.

### 2.4. Microsomal Stability

As **Dia2** showed a good DOT1l inhibition, its stability upon exposure to mouse liver microsomes was evaluated and compared to parent compounds EPZ-4777 **1** and EPZ-5676 **2**. As mentioned in the literature [26], the metabolic stability of the Epizyme’s molecules is low with a half-life of 5.3 min and 5.7 min, respectively. Compound **Dia2** also showed high clearance but the structural modifications induced a 2-fold half-life increase to 11.5 min (intrinsic clearance of 120.6 µL/min/mg).

### 2.5. Molecular Docking

**Figure 3:**
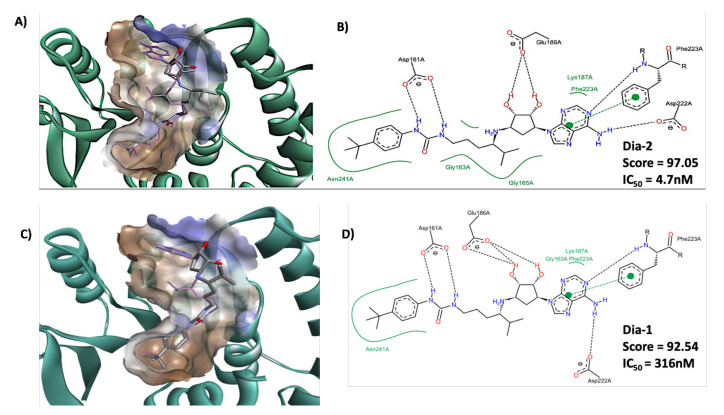
Representation of the docking of **Dia1** and **Dia2** using the crystal structure of DOT1L with EPZ-4777 **1**(PBD 4ER3). (**A**) Representation of compound **Dia2** inside the catalytic pocket of human DOT1L, the hydrophobicity surface being represented by a cloud going from blue (hydrophilic zone) to brown (more hydrophobic zone) magenta sticks represents co-crystal ligand. (**B**) Representation of the interacting amino acid of DOT1L catalytic pocket with **Dia2**. (**C**) Representation of **Dia1** inside the catalytic pocket of human DOT1L, the hydrophobicity surface being represented by a cloud going from blue (hydrophilic zone) to brown (more hydrophobic zone). Magenta sticks represent co-crystal ligand. (**D**) Representation of the interacting amino acids of DOT1L catalytic pocket with Dia1.

To explain the difference in the inhibitory activity of the two diastereoisomers Dia1 and Dia2, we performed a molecular docking study with the GOLD 5.8.1 software (Supplementary Figure S1). The crystal structure of DOT1L in complex with EPZ-4777 **1** (PDB: 4ER3) was used, as **Dia1** and **Dia2** are structurally similar to compound **1** and, thus, we assumed could present a similar binding mode [16]. The docking results showed that for the best-ranked conformations, compounds **Dia1** and **Dia2** adopt the similar pose and orientation observed for the cocrystal with the EPZ-4777 inhibitor (Figure 3A,C). Moreover, the adenosinylcyclopentandiol moiety in compounds **Dia1** and **Dia2** establish the same type of interactions as the adenosine moiety of the cofactor SAM inside the catalytic pocket. The urea linker in compounds **Dia1** and **Dia2** forms a hydrogen bond with Asp161, similarly as in EPZ-4777 **1**. Interestingly, the docking of the two diastereoisomers in the catalytic pocket showed that the isopropyl group of **Dia2** interacts with amino acids Gly163 and Gly165 via nonpolar interactions, whereas the isopropyl group in **Dia1** is flanking out of the catalytic pocket. Overall, these observations are supported by the different Gold Score calculated after docking for compounds **Dia1** (GoldScore = 92.5456) and **Dia2** (GoldScore = 97.0547) [27]. We hypothesize that the single inversion of the stereocenter in the linker and different orientation of the isopropyl group between **Dia1** and **Dia2** contributes to the different inhibitory profiles, in agreement with the IC_50_ values measured against DOT1L (Figure 2).

### 2.6. **Dia2** Inhibits Cell Proliferation by a Cytostatic Effect

**Figure 4:**
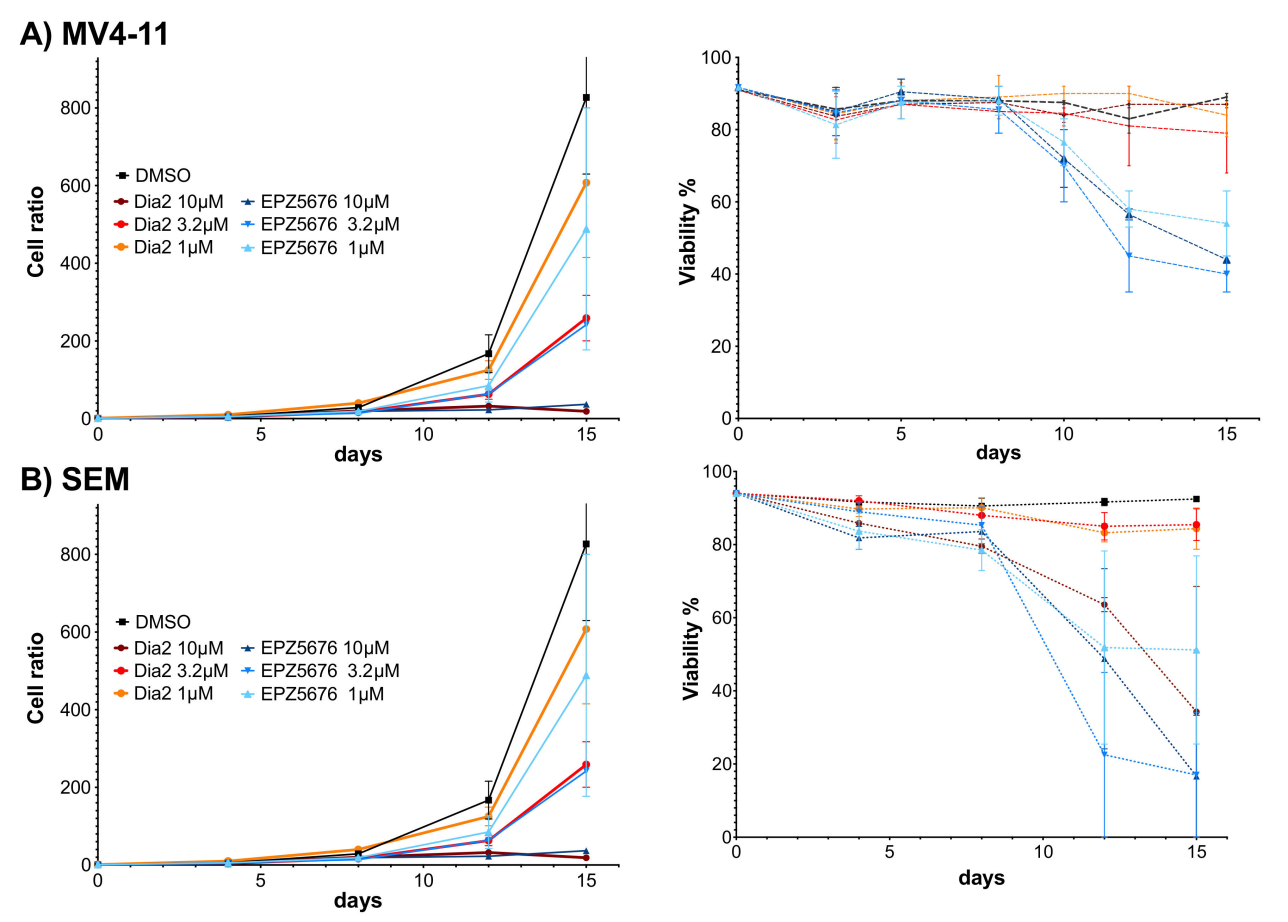
**MLL-AF4 cells: MV4-11** (**A**) **and SEM** (**B**). Right, growth of MLL-AF4 cells up to 15 days of incubation with 10 µM (bordeaux red circles), 3.2 µM (red circles), 1 µM (orange circles) of **Dia2**, and 10 µM (dark blue triangles), 3.2 µM (blue triangles), 1 µM (sky blue triangles) of EPZ-5676 compared to DMSO (black squares) were plotted as the ratio of cell amount over initial population. Left, viable cells were counted every 3 to 4 days in the presence of **Dia2**, EPZ-5676, or DMSO, the percentage of viability (dotted curves) is reported. Every data point is the result of technical triplicates and biological duplicates.

**Figure 5:**
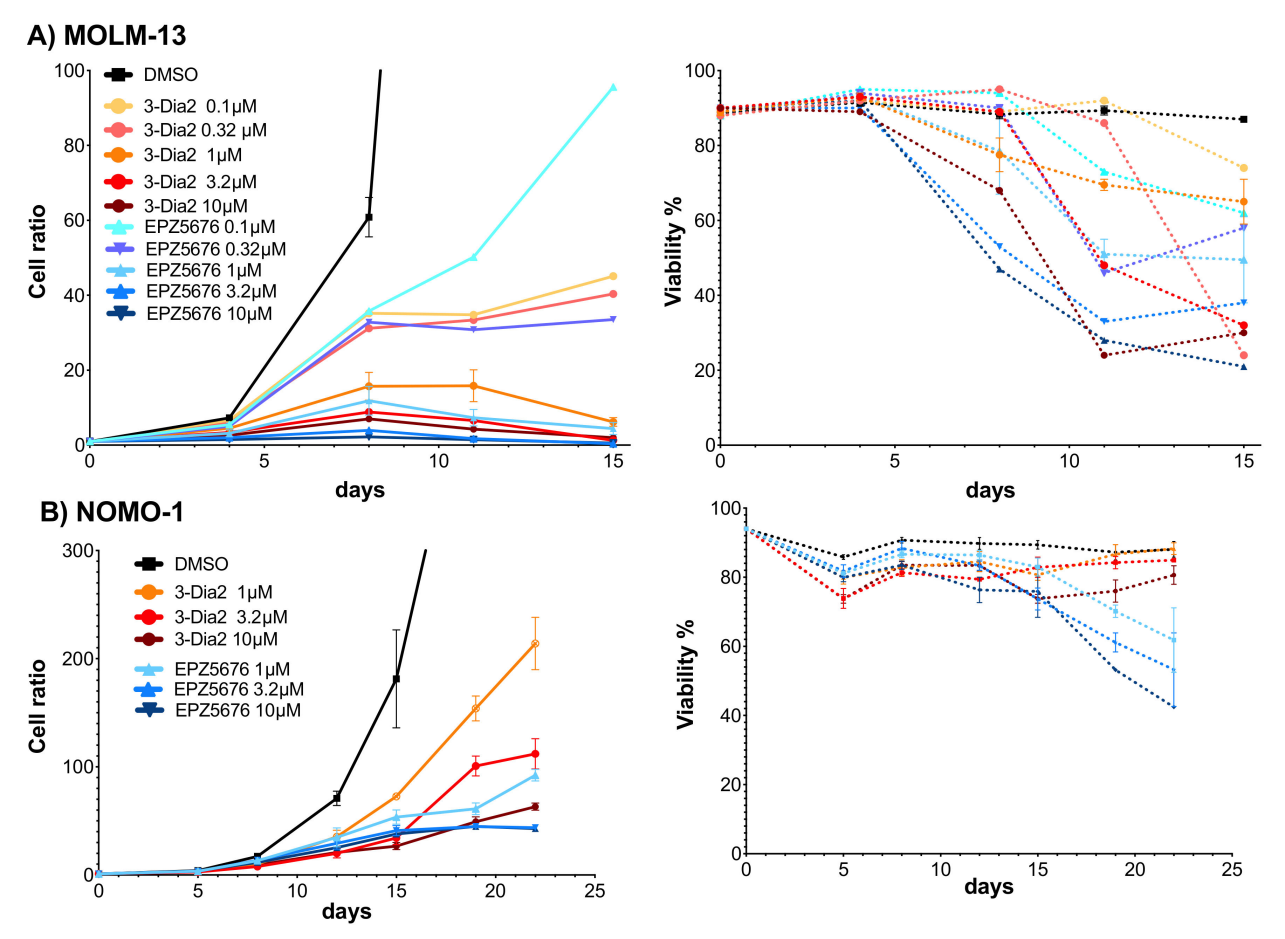
**MLL-AF9 cells: MOLM13 and NOMO-1.** (**A**) Right, growth of MOML-13 (MLL-AF9) cells up to 15 days of incubation with 3.2 µM (red circles), 1 µM (orange circles), 0.32 µM (pink circles) of **Dia2**, and 3.2 µM (cyan triangles), 1 µM (sky blue triangles), 0.32 µM (violet triangles) of EPZ-5676 compared to DMSO treated cells (black squares) were plotted as the ratio amount of cells over initial population. Left, viable cells were counted every 3 to 4 days in the presence of **Dia2**, EPZ-5676, or DMSO, and the percentage of viability is reported as dotted curves. Every data point is the result of technical triplicates (**B**) Right, growth of NOMO-1 cells (MLL-AF9) up to 22 days of incubation with 10 µM (bordeaux red circles), 3.2 µM (red circles), 1 µM (orange circles) of **Dia2**, and 10 µM (dark blue triangles), 3.2 µM (cyan triangles), 1 µM (sky blue triangles) of EPZ-5676 compared to DMSO-treated cells (black squares) were plotted as the ratio amount of cells over initial population. Left, viable cells were counted every 3 to 4 days in the presence of **Dia2**, EPZ-5676, or DMSO, and the percentage of viability is reported as dotted curves. Every data point is the result of biological duplicates.

**Figure 6:**
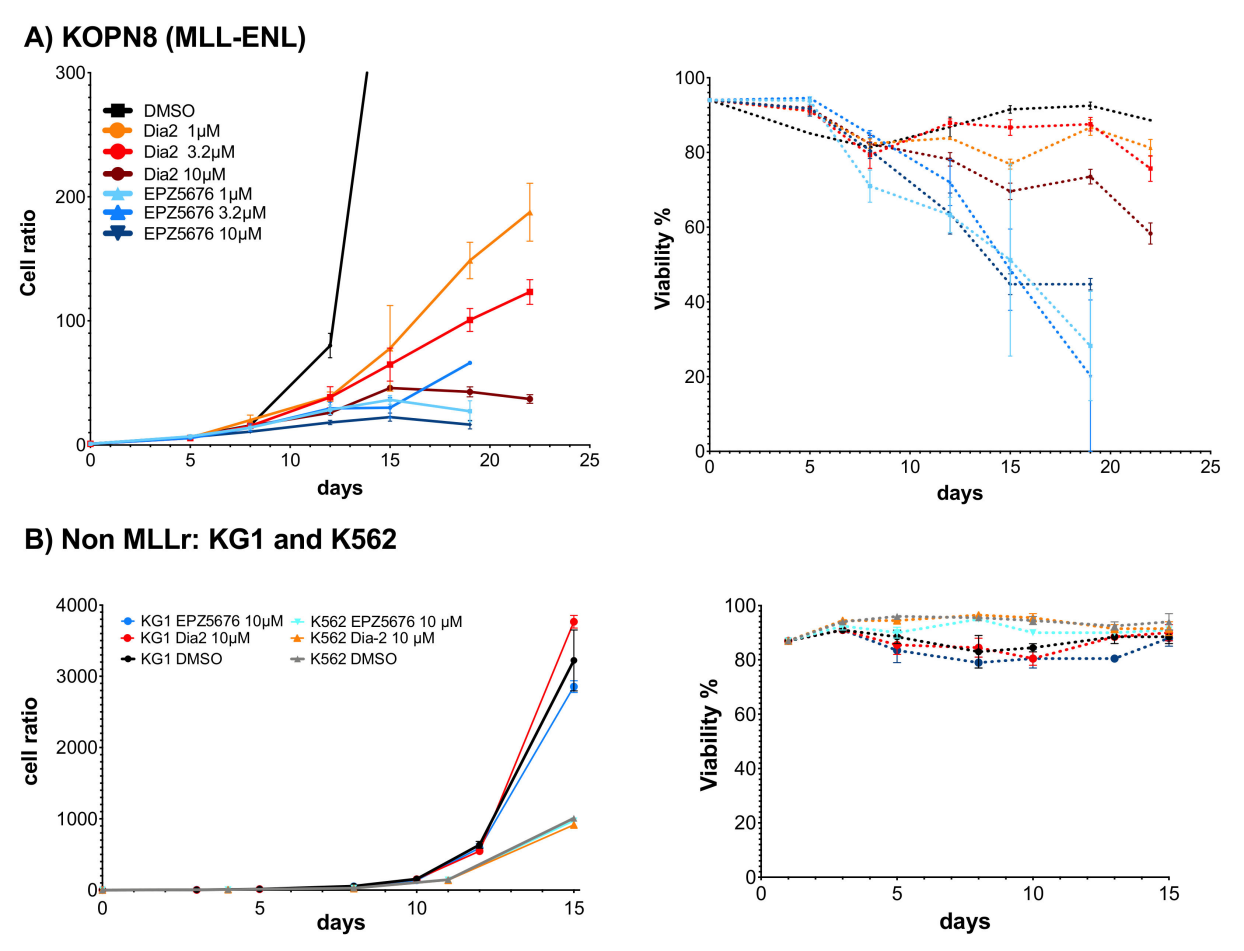
(**A**) Right, growth of KOPN8 cells (MLL-ENL) up to 22 days of incubation with 10 µM (bordeaux red circles), 3.2 µM (red circles), 1 µM (orange circles), of **Dia2** and 10 µM (dark blue triangles), 3.2 µM (cyan triangles), 1 µM (sky blue triangles) of EPZ-5676 compared to DMSO treated cells (black squares) were plotted as the ratio amount of cells over initial population. Left, viable cells were counted every 3 to 4 days in the presence of **Dia2**, EPZ-5676, or DMSO, and the percentage of viability is reported as dotted curves. Every data point is the result of technical triplicates and biological duplicates. (**B**) Right, growth of non MLLr leukemia cells KG1 (circles) and K562 (triangles) up to 15 days of incubation with 10 µM (red and orange) of **Dia2** and 10 µM (blue and cyan) of EPZ-5676 compared to DMSO treated cells (black and grey) were plotted as the ratio amount of cells over initial population. Left, viable cells were counted every 3 to 4 days in the presence of **Dia2**, EPZ-5676, or DMSO, and the percentage of viability is reported as dotted curves. Every data point is the result of technical triplicates and biological duplicates.

As **Dia2** is a nanomolar inhibitor of DOT1L and shows improved metabolic stability, it was tested in MLL-r cells for its ability to inhibit cell proliferation and was compared to EPZ-5676, the most potent DOT1L inhibitor in cells [28]. MLL-AF4 cells, here MV4-11, were cultured with **Dia2** or EPZ-5676 at different concentrations. Cell growth and viability were measured every three days in parallel to an unexposed test population. Cells were re-seeded in a controlled number after each count, in a new medium containing the inhibitor at the same concentration, in order to maintain an optimal growth medium (Figure 4, Figure 5 and Figure 6).

Little effect was observed during the first days of exposure, which was consistent with the fact that the concentrations are far from the LD50. This analysis also revealed a significant delay before the anti-proliferative effects of **Dia2** and EPZ-5676 appeared, as MV4-11 cells continued to proliferate at normal levels for several days after exposure to the inhibitor (Figure 4A). This reflects the time required to completely reverse the aberrant expression of the MLL fusion target genes upon inhibition of DOT1L, a process that involves depletion of methylated H3K79, followed by a decrease in mRNA expression and reduced levels of gene products critical for the growth of leukemic cells [28]. During 8 days, the viability of the cells remained comparable to non-treated cells. After 8 days, MV4-11 cell viability decreased by half upon treatment with reference compound EPZ-5676, inducing a cytotoxic effect. In contrast, treatment with **Dia2** affected cell proliferation without affecting cell viability, showing a cytostatic effect. MV4-11 cells remained viable in the presence of **Dia2**, but their numbers remained constant indicating that they had stopped dividing.

Next, the effect of **Dia2** treatment was evaluated on other MLLr cells. We found that **Dia2** had the ability to prevent the proliferation of MOLM-13 cells (MLL-AF9 rearranged cells) in vitro even at very low concentrations (Figure 5A). Noteworthy, while both compounds showed similar effects at high concentrations, **Dia2** showed more lasting and significant effects at the lower concentrations. This suggests that **Dia2** is either more active or more difficult to metabolize by the cells [26]. The effect of the compound **Dia2** was tested in other MLL-r cell lines (SEM (Figure 4B), NOMO-1 (Figure 5B), and KOPN8 (Figure 6A)) during 15 days of treatment (Table 1). Interestingly the results confirmed that **Dia2** is as potent as EPZ-5676 to decrease cell proliferation but shows an interesting cytostatic behavior. In addition, it shows that the effect is independent of the MLL fusion partner, as KOPN8 cells have translocation t(11;19) and expresses the MLL-ENL fusion gene, MOLM13 and NOMO-1 cells have translocation t(9;11) and express MLL-AF9, whereas MV4-11 and SEM cells have translocation t(4;11) and express MLL-AF4 fusion gene (7). Interestingly, **Dia2** ability to inhibit cell proliferation in MLLr cells parallels the one of EPZ5676 and is dependent on the cell line.

Finally, **Dia2** showed no effect on cell proliferation and viability in two non-MLLr leukemia lines (KG-1 and K562) even at high concentrations (Figure 6B), confirming its specificity for MLL-r cells.

### 2.7. Dia2 Inhibits Cellular H3K79 Dimethylation

**Figure 7:**
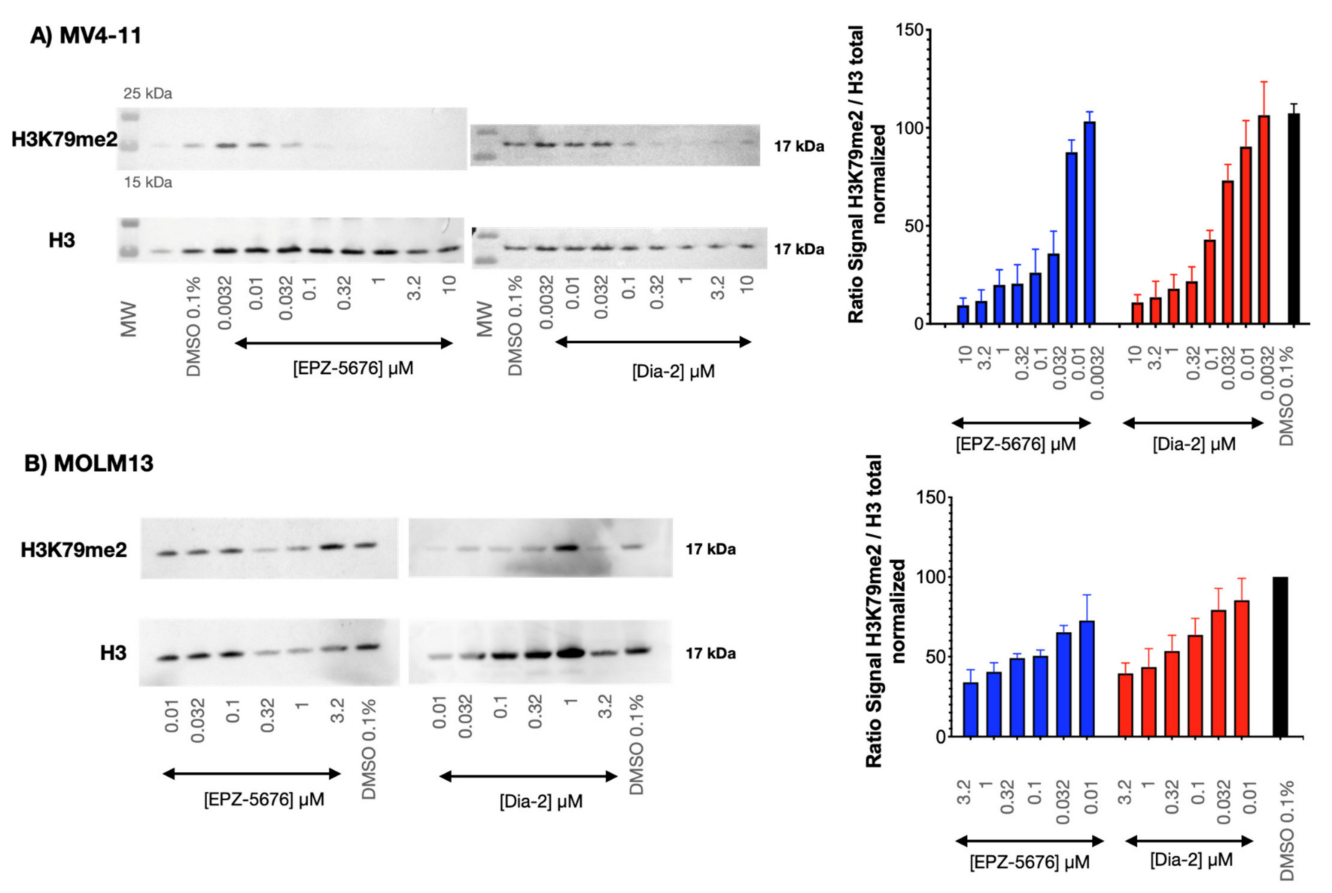
(**A**) **MV4-11** and (**B**) **MOLM13.** The percentage of dimethylated H3K79 lysine measured by Western blot of harvested histones of MV4-11 cells after treatment with **Dia2** (red) and EPZ-5676 (in blue). A representative Western blot is shown as an example. The values are the mean results of at least biological triplicated and correspond to the normalization to the H3K79me2 signal to the histoneH3 signal, as a percentage of methylation compared to DMSO-treated cells. MV4-11 cells were treated 4 days and MOLM-13 6 days.

Having established that **Dia2** is a potent and highly selective inhibitor of DOT1L in biochemical assays and inhibits cell proliferation specifically of MLLr cell lines, we explored the ability of **Dia2** to inhibit DOT1L in MLLr cells and decrease the methylation of H3K79. Western blot analysis of the H3K79me2 normalized to the total H3 of MV4-11 (Figure 7A) cells treated with increasing concentrations of **Dia2** and EPZ-5676 show an efficient decrease in methylation compared to DMSO treated cells (Figure 7A). **Dia2** and EPZ-5676 are both similarly efficient at inhibiting DOT1L in cells. Next, we evaluated the effect on the demethylation of H3K79 in MLL-AF9 MOLM13 cells (Figure 7B). Again, **Dia2** was as efficient as EPZ-5676 to inhibit the H3K79me2 mark.

### 2.8. Dia2 Inhibits Cellular MLL Target Genes and Causes Cell Cycle Change and Apoptosis in MLL-Rearranged Cells

**Figure 8:**
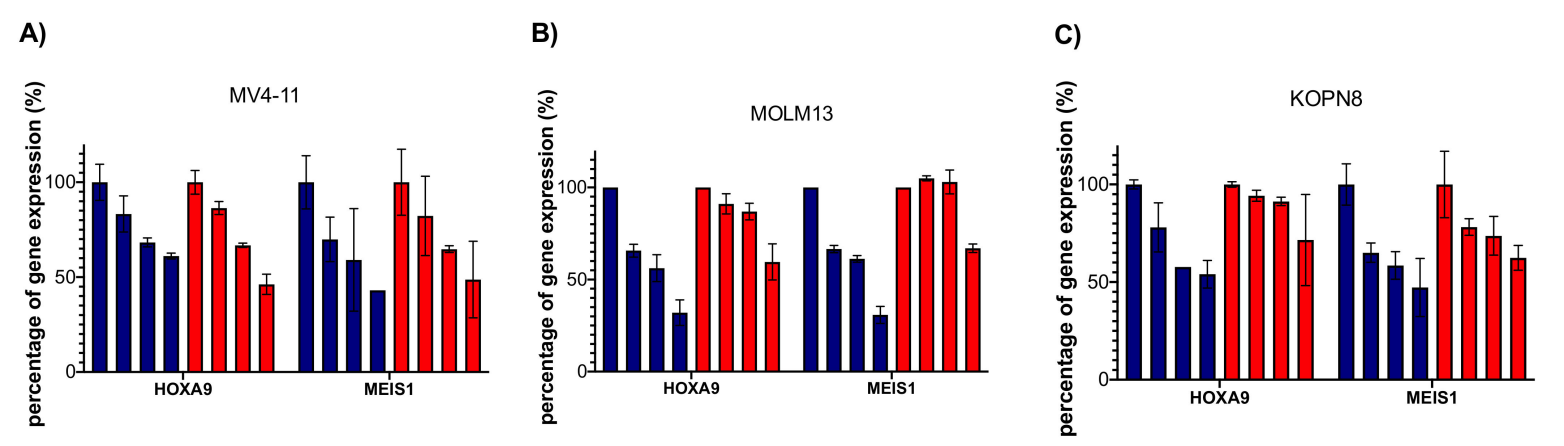
Expression variation of HOXA9 and MEIS1 genes in MV4-11 cells (**A**), MOLM-13 (**B**), and KOPN8 (**C**) treated for 6 days and 10 days, respectively, in the presence of **Dia2** (in red) or EPZ-5676 (in blue) at the indicated concentration. The expression of each gene is quantified by RT qPCR relative to the B2M reference gene for HOXA9 and YWAX for MEIS-1. Data points result in mean normalized values issued from technical triplicates and biological duplicates.

We then investigated the impact of the inhibition of H3K79me2 in MLLr cells on the expression of the pro-leukemic genes, HOXA9 and MEIS1. These are MLL target genes and have been characterized for being activated by the aberrant H3K79 methylation by DOT1L recruited at these genes by the MLL-fusion proteins [29,30,31] and inhibited by DOTL1 inhibitors [15,17]. By quantitative real-time PCR, we observed that increasing concentrations of **Dia2** (0.1, 0.32, 1 and 3.2 µM, red bars,) led to a decrease in a dose-dependent manner of MEIS1 and HOXA9 expression in MV4-11 (Figure 8A), MOLM13 (Figure 8B) and KONP8 (Figure 8C), after 6 days of incubation for the first two cell lines and 10 days for the latter. The difference in kinetics is related to the doubling time of the cells (Table 1), indeed, we measured that the doubling time of MV4-11 is 12 h while the doubling time for MOLM-13 is 22 h. The efficacy of **Dia2** is comparable to EPZ-5676 (blue bars).

These results, together with the inhibition of MLLr cell proliferation are encouraging, and thus we next studied the effect of the compound on the cell cycle and apoptosis.

**Figure 9:**
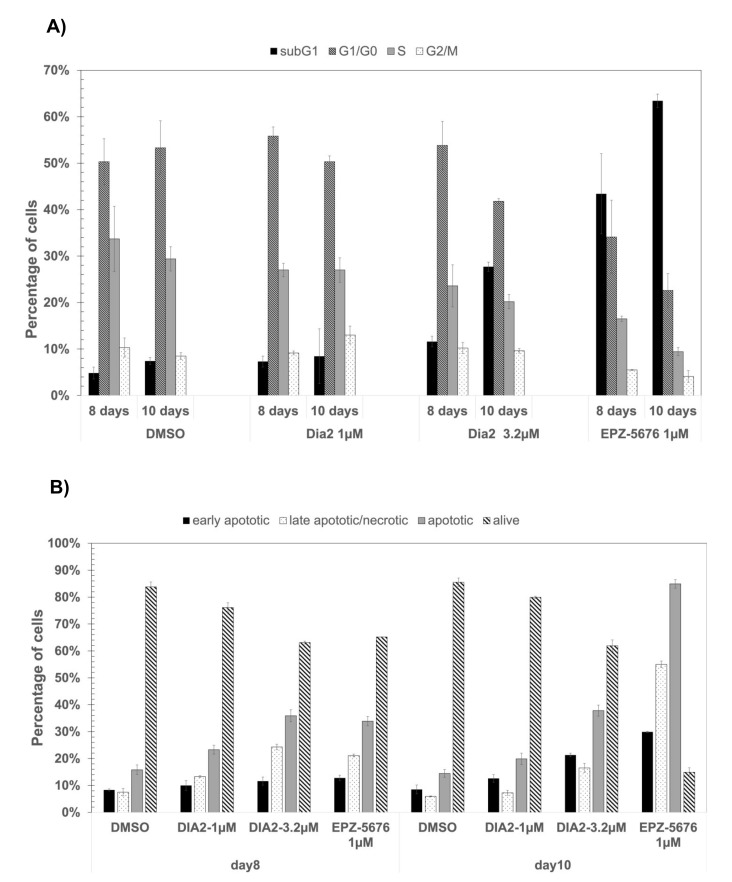
Cell cycle and apoptosis analysis of **MV4-11** cells treated with **Dia2**. MV4-11 cells with 1 µM or 3.2 µM **Dia2** and 1 µM EPZ-5676. The effect on the cell cycle (**A**) and apoptosis (**B**) over time was measured by staining for DNA content and Annexin V using flow cytometry. Cells treated with an equal concentration of DMSO were used as negative controls. The error bars indicate the difference between the two replicates in the experiment.

In order to explore the mechanism of cell proliferation inhibition caused by **Dia2**, we performed flow cytometry experiments to measure the effects of **Dia2** on the cell cycle and apoptosis in MV4-11 cells for DNA content and Annexin V staining (Figure 9 and Supplementary Figures S3 and S4). As shown in Figure 9A and Supplementary Figure S3, treatment with **Dia2** at 1 µM does not affect the cell cycle of MV4-11 cells, the DNA content is practically the same compared with control cells treated with DMSO. However, treatment with 3.2 µM of **Dia2** led to a decrease in the percentage of cells in the G0/G1 phase from 53% (day 8) to 41% (day 10), 24% (day 12) in a time-dependent manner. A decrease in the S phase of the cell cycle was also observed from 24% (day 8) to 20% (day 10), 13% (day 12). Interestingly, **Dia2** induced very little apoptosis (as shown in Figure 9B and Supplementary Figure S4): only a slight increase in Annexin V positive cells was observed from day 8 to day 10 when the concentration increased from 1 µM (apoptotic: 23%-day 8, 20%-day 10) to 3.2 µM (apoptotic: 35%-day 8, 38%-day 10). These results confirm that **Dia2** arrests cell proliferation (Figure 4, Figure 5 and Figure 6) without inducing cell death differently from EPZ-5676 that induces an increase in cells in subG1 and an increase in apoptosis (Figure 9).

## 3. Discussion

DOT1L has emerged as an attractive therapeutic target in MLLr. The majority of MLL rearrangements result in the expression of MLL fusion proteins that gain the ability to recruit DOT1L directly or indirectly to MLL target genes. This leads to inappropriate hypermethylation of H3K79 and increased expression of a panel of target genes, including HOXA9 and MEIS1, which promote leukemogenesis.

We designed an analog of the described selective DOT1L inhibitor EPZ-4777 **1**, aiming at minimizing several extrapolated metabolic weaknesses. Two diastereoisomers **Dia1** and **Dia2** were synthetized and tested against DOT1L. Diastereoisomer **Dia2** was 67-fold more potent at inhibiting the enzymatic activity of DOT1L in vitro (IC_50_ of 4.7 nM similar to the parent compound EPZ-4777 **1**), highlighting the role of the additional stereocenter. Moreover, the structural modifications allowed to slightly increase the metabolic stability in mouse liver microsomes as **Dia2** showed a half-life of 12 min when EPZ derivatives were approximatively 5 min. Clearance of Dia2 remains high, but hydroxylation of the tert-butyl group was spotted as an important metabolite. Pharmacomodulation of the *tert*-butyl group would be a future key step in order to improve metabolic stability. Molecular docking of the diastereomers **Dia1** and **Dia2** in the crystal structure of DOT1L (PDB: 4ER3) suggested that the difference in the inhibitory profile is due to the orientation of the isopropyl group in **Dia1** away from the catalytic pocket and thus subsequent weaker affinity compared to **Dia2**.

As **Dia2** showed an increased microsomal stability compared to the most cell-active DOT1L inhibitor EPZ-5676, we tested its effect on the proliferation of leukemia cell lines. Interestingly, long treatment with low doses of **Dia2** arrest cell proliferation specifically in MLLr cells carrying MLL-AF9, MLL-ENL, and MLL-AF4 fusions and not in other leukemia cells, as observed with EPZ-5676. More interestingly, this effect is related to a cytostatic effect of the compound with little cell death. This is very different from what is observed with EPZ-5676 that induces cell death. This difference in the mechanism was further confirmed by flow cytometry analysis of the impact of the compounds on the cell cycle and apoptosis. Surprisingly, this difference is not related to the ability to inhibit DOT1L in cells, as both compounds inhibit efficiently H3K79 dimethylation in MV4-11 and MOLM13 cells with similar EC_50_ in the microM range. In agreement, both compounds decreased similarly the expression of the pro-leukemic genes HOXA9 and MEIS-1 in a dose-dependent manner on the sixth day of treatment for MV4-11 and MOLM-13 and on the tenth day for KOPN8. Furthermore, **Dia2** is selective of the H3K79me2 mark in cells and does not affect the other 7 tested histone marks (H2R2me2S, H3K4me3, H3K9me2, H4K20me2, H4K20me3, H3K9me1, H3K36me1 (Supplementary Figure S4). It was also unable to inhibit the catalytic activity of human DNMT3A, and HMTs EZH2 complex, PRMT4, PRMT6, SET7/9, and SMYD2 (Supplementary Table S1).

Thus, the difference in cellular activity could be linked to the difference in metabolism for the two compounds as it was the starting hypothesis for the design of compounds **3**. Indeed, according to the literature, DOT1L knock-down has a minimal effect on cancer cell growth [28,32,33], while it plays an essential role during development and embryogenesis [28,34]. Compound **Dia2** has been designed to avoid the fragile points of metabolization of the Epizyme derivatives and thus the difference in metabolites could explain the difference in cellular activity and the decrease in the cytotoxicity. In addition, while no demethylases have been identified for H3K79me1/2/3, it has been shown that nucleosome turnover plays an important role in the regulation of the methylation of this mark [35]. Thus, the cellular activity of Dia2 could be linked to the inhibition of DOT1L at different steady-state levels of H3K79 methylation in respect of EPZ-5676, explaining the difference in the observed effect.

Importantly, as the precise function of DOT1L in adult cells remains to be elucidated, this unique feature of **Dia2** is of most interest as it can be exploited as a chemical probe to study the impact of the DOT1L inhibition and the decrease in the corresponding H3K79 methylation mark without inducing cell death.

Another important observation is the fact that the time at which the effect of the compound appears is correlated to the doubling time of the cell line studied (Table 1). Indeed, this is in agreement with specific cellular inhibition of DOT1L by **Dia2**, as it acts by a passive demethylation mechanism. Inhibition of DOT1L activity prevents the enzyme from catalyzing new methylations and the methylation already present is diluted over the course of cell divisions. Nucleosome turnover is particularly important for the demethylation of H3K79 as no demethylase has been identified [32]. In addition, Table 1 shows that the MLLr cell lines have a different sensitivity to DOT1 inhibition by both **Dia2** and EPZ5676.

In conclusion, we developed here a novel potent inhibitor of DOT1L, **Dia2**. The compound constitutes a starting point for improved metabolic resistance. In addition, **Dia2** exhibits selectivity towards DOT1L vs. other HMTs and DNMT3A. The selectivity of **Dia2** antiproliferative activity for MLL rearranged cell lines is also encouraging from the point of view of the development of DOT1L inhibitors as targeted therapies.

Further development needs to be carried out to improve the potency of the molecule and metabolic properties, as DOT1L has been reported to be a target of interest in diverse diseases [34]. In addition to its known role in MLLr leukemia, DOT1L plays a part in other cancers such as prostate cancer and multiple myeloma [34]. DOT1L was also shown to be essential for the growth and viability of a subset of multiple myeloma cell lines along with the SETDB1 protein [35]. DOT1L is found significantly increased in gastric malignant tumors and regulates cyclin-dependent kinase (CDK) 4 and 6, which accelerate the proliferation of the gastric tumors [36]. Moreover, DOT1L orthologs were also found in other organisms such as *Ornithodoros moubata* [37] that is responsible for the African swine fever virus. This protein is overexpressed in the first stage of life of the parasite and the chemical inhibition of it leads to the death of the organism, making this DOT1L analog protein a great target for the treatment of the disease Moreover, H3K79me2 increases progressively with aging, suggesting its role in aging; therefore, DOT1L seems to be also an attractive target for diseases associated with aging deregulation, such as Hutchinson-Gilford progeria syndrome [38].

Finally, at the state of the art, **Dia2** is the first DOT1L inhibitor that efficiently decreases H3K79me2 and inhibits cell proliferation by a cytostatic effect making it an optimal chemical probe to study the cellular consequences of the inhibition of DOT1L devoid of cell cytotoxicity as it is the case of EPZ-5676.

## 4. Materials and Methods

### 4.1. Chemical Synthesis

All chemicals were from Sigma-Aldrich (St Quentin-Fallavier, France), Alfa Aesar (Thermofisher Scientific, Les Ulis, France), Carbosynth (Compton, UK), and FluoChem (Hadfield, UK). NMR experiments were recorded on an Agilent DirectDrive 500 spectrometer (Agilent Technologies, Les Ulis, France) with a proton resonating frequency of 500 MHz. Some NMR experiments were recorded on a 600 MHz Avance NEO (Bruker Biospin, Palaiseau, France) spectrometer with a 14.1 Tesla magnetic field. The spectrometer was equipped with a cryogenically cooled triple resonance 1H [13C/15N] probe. Spectra were recorded using TopSpin 4.07 (Bruker Biospin). Spectra were recorded using VnmrJ 4.2A (Agilent Technologies, Santa Clara, CA, USA). Chemical shifts are given in ppm. Coupling constants *J* are measured in Hz. Splitting patterns are designed as follows: s, singlet; bs broad singlet; d, doublet; bd broad doublet; t, triplet; brt, broad triplet; dd, doublet of a doublet; m, multiplet; ddd, doublet of a doublet of a doublet; q, quartet; quint, quintet, MS-ESI were obtained on a Bruker MicroTOF. HRMS analyses were performed on a Q Exactive mass Spectrometer (Thermofisher, Les Ulis, France) using direct injection. Samples were previously dissolved in a mix of water and acetonitrile (50/50) and 0.1% of formic acid. Full scans (150–2000 Da) were acquired in positive ion mode with a resolution of 70,000.

**2-Methyl-5-hexen-3- *tert*-butyldiphenylsilyl ether (4)**

To a solution of 2-methyl-5-hexen-3-ol (2.0 g, 17.5 mmol), imidazole (2.4 g, 70 mmol) in DMF (50 mL), TBDPSCl (4.8 mL, 17.5 mmol) was added. The reaction mixture was stirred at room temperature overnight and then the volatiles were removed under high vacuum. The residue was purified by silica gel flash chromatography using cyclohexane as eluent. Compound **1** was obtained as a clear oil (6.0 g, 17.1 mmol, 96%).

**MS-ESI(m/z)** calculated for C_23_H_33_OSi [M+H]^+^: 353.2; Found: 353.3.

**^1^H NMR** (500 MHz, chloroform*-d*) δ 7.75–7.68 (m, 4H), 7.48–7.41 (m, 2H), 7.44–7.35 (m, 4H), 5.63 (ddt, *J =* 17.4, 10.4, 7.1 Hz, 1H), 4.93–4.87 (m, 1H), 4.87 (ddt, *J =* 12.7, 2.2, 1.2 Hz, 1H), 3.61 (ddd, *J =* 6.9, 5.6, 3.7 Hz, 1H), 2.18 (tddd, *J =* 14.1, 11.2, 7.0, 5.7 Hz, 2H), 1.75 (pd, *J =* 6.9, 3.6 Hz, 1H), 1.08 (d, *J =* 5.9 Hz, 1H), 1.08 (s, 8H), 0.89 (dd, *J =* 23.3, 6.8 Hz, 5H).

**^13^C NMR** (126 MHz, chloroform-*d*) δ 136.0, 135.5, 134.7, 134.3, 129.4, 129.3, 127.4, 127.3, 116.3, 38.3, 32.0, 27.1, 19.5, 18.7, 16.7.

**(*tert*-Butyldiphenylsilyl)oxy]-5-methylhexan-1-ol (5)**

To a solution of **4** (6.0 g, 17.0 mmol) in THF (60 mL) was added dropwise a 1 M solution of boran–DMS complex in THF (5 mL). The mixture was stirred at room temperature for 4 h. Then a mixture of hydrogen peroxide 30% (20 mL) and 1M sodium hydroxide (40 mL) was added dropwise at 0 °C and the solution was stirred at room temperature overnight. The crude was then portioned between ethyl acetate and water, the organic phase was dried with brine and anhydrous sodium sulphate. The solvents were evaporated, and the residue was purified by silica gel flash chromatography using a linear gradient of 0–10% ethyl acetate in cyclohexane. Compound **5** (4.5 g, 12.2 mmol, 71%) was obtained as a clear oil.

**MS-ESI(m/z)** calculated for C_23_H_35_O_2_Si [M+H]^+^: 371.2; Found: 371.2.

**^1^H NMR** (500 MHz, chloroform*-d*) δ 7.72 (td, *J =* 5.0, 4.5, 2.3 Hz, 4H), 7.48–7.42 (m, 2H), 7.40 (t, *J =* 7.6 Hz, 4H), 3.58 (d, *J =* 4.5 Hz, 1H), 3.38 (d, *J =* 5.7 Hz, 2H), 1.80 (pd, *J =* 6.9, 4.1 Hz, 1H), 1.43 (ddd, *J =* 11.2, 8.5, 6.0 Hz, 3H), 1.37 (dd, *J =* 10.3, 7.0 Hz, 1H), 1.09 (s, 9H), 1.04 (s, 1H), 0.94 (d, *J =* 6.8 Hz, 3H), 0.85 (d, *J =* 6.8 Hz, 3H).

**^13^C NMR** (126 MHz, Chloroform*-d*) δ 136.0, 134.8, 134.4, 129.4, 127.4, 63.0, 32.5, 28.8, 27.1, 19.6, 18.2, 17.5.

**2-(4-((*tert*-Butyldiphenylsilyl)oxy)-5-methylhexyl)isoindoline-1,3-dione (6)**

A solution of **5** (1.0 g, 2.7 mmol), phthalimide (0.6 g, 6.0 mmol) and triphenylphosphine (1.1 g, 6.0 mmol) in THF (40 mL) was stirred under argon at room temperature for 30 min. Then, the solution was cooled at 0 °C, DIAD was added dropwise and stirred at 0 °C for over 15 min until the yellow color persisted. The mixture was stirred overnight. Tetrahydrofuran was evaporated and the crude portioned between ethyl acetate and a 1M NaHCO_3_ solution. The organic phase was washed with water and brine then dried over anhydrous magnesium sulphate. The solvent was evaporated, and the residue was purified by silica gel flash chromatography using a linear gradient of 0–50% ethyl acetate in cyclohexane as eluent. The product **6** (1.3 g, 2.6 mmol, 93%) was obtained as a clear oil.

**MS-ESI(m/z)** calculated for C_31_H_38_NO_3_Si [M+H]^+^: 500.2; Found: 500.2.

**^1^H NMR** (500 MHz, chloroform*-d*) δ 7.84 (dd, *J =* 5.5, 3.0 Hz, 2H), 7.78–7.70 (m, 2H), 7.73–7.65 (m, 4H), 7.44–7.31 (m, 7H), 3.56 (td, *J =* 5.6, 4.0 Hz, 1H), 3.47 (td, *J =* 7.4, 6.8, 1.3 Hz, 2H), 1.76 (m, 1H), 1.66–1.47 (m, 2H), 1.49–1.41 (m, 2H), 1.41 (dd, *J =* 8.0, 1.8 Hz, 1H), 1.12–1.05 (m, 1H), 1.07 (s, 9H), 0.91 (d, *J =* 6.8 Hz, 3H), 0.83 (d, *J =* 6.9 Hz, 3H).

**^13^C NMR** (126 MHz, chloroform*-d*) δ 168.2, 136.0, 134.7, 134.3, 133.8, 132.2, 129.4, 129.4, 127.4, 127.4, 123.1, 77.4, 37.9, 32.4, 30.2, 27.1, 24.6, 19.5, 18.2, 17.5.

**5-Methyl-6-((t*ert*-butyl)-diphenyl silylether) hexanamine (7)**

A solution of **6** (1.3 g, 2.6 mmol) in 20 mL of a 10% solution of methyl hydrazine in methanol was stirred at room temperature overnight. The solvent was evaporated, and the residue was solubilized in ethyl acetate. The organic layer was washed with water then brine and dried over anhydrous magnesium sulphate. The solvent was evaporated, and the residue was purified by silica gel flash chromatography using a linear gradient of 0–10% methanol in dichloromethane as eluent. The compound **7** (0.8 g, 2.2 mmol, 81%) was obtained as a clear oil that solidified over time.

**MS-ESI(m/z)** calculated for C_23_H_36_NOSi [M+H]: 370.2; found: 370.3

**^1^H NMR** (500 MHz, Chloroform*-d*) δ 7.75–7.68 (m, 4H), 7.48–7.34 (m, 6H), 3.57 (td, *J =* 5.8, 3.8 Hz, 1H), 2.42 (t, *J =* 7.0 Hz, 2H), 1.78 (td, *J =* 6.8, 3.7 Hz, 1H), 1.44–1.36 (m, 1H), 1.39–1.30 (m, 1H), 1.25 (m, 2H), 1.20 (s, 2H), 1.08 (s, 9H), 0.94 (d, *J =* 6.9 Hz, 3H), 0.86 (d, *J =* 6.9 Hz, 3H).

**^13^C NMR** (126 MHz, Chloroform*-d*) δ 136.0, 134.9, 134.4, 129.3, 127.3, 42.2, 32.4, 30.3, 27.1, 19.6, 18.3, 17.2.

**1-(4-(*tert*-Butyl)phenyl)-3-(4-((*tert*-butyldiphenylsilyl)oxy)-5-methylhexyl)urea (8)**

To a solution of 4-nitrophenylchloroformate (326 mg, 2.4 mmol) in dichloromethane (5 mL) was added 4-dimethylaminopyridine (200 mg, 2.4 mmol). The mixture was stirred under argon at room temperature. A solution of t*ert*-butylaniline (250 µL, 2.4 mmol) in dichloromethane (5 mL) was added dropwise over 10 min and the mixture was stirred at room temperature for another 10 min. A solution of **7** (400 mg, 1.1 mmol) in dichloromethane (2 mL) was added and stirred for approximately 1 h (conversion followed by TLC using dichloromethane as eluent). The mixture was diluted with dichloromethane and washed three times with a 1M potassium carbonate solution, then with a 1M citric acid solution, water, and brine. The organic phase was dried over anhydrous magnesium sulphate. The solvent was evaporated, and the residue was purified by silica gel flash chromatography using dichloromethane as eluent. The compound **8** (400 mg, 1.1 mmol, 65%) was obtained as a white solid.

**MS-ESI(m/z)** calculated for C_34_H_49_N_2_O_2_Si [M+H]^+^: 544.3; Found: 544.3.

**^1^H NMR** (500 MHz, Chloroform*-d*) δ 7.70 (ddt, *J =* 6.4, 4.8, 1.5 Hz, 4H), 7.46–7.32 (m, 8H), 7.19–7.12 (m, 2H), 6.15 (s, 1H), 4.40 (t, *J =* 5.6 Hz, 1H), 3.56 (td, *J =* 5.4, 3.8 Hz, 1H), 3.04–2.86 (m, 2H), 1.77 (pd, *J =* 6.8, 3.9 Hz, 1H), 1.42–1.31 (m, 3H), 1.33 (s, 9H), 1.31–1.23 (m, 1H), 1.07 (s, 8H), 0.92 (d, *J =* 6.8 Hz, 3H), 0.85 (d, *J =* 6.8 Hz, 3H).

**^13^C NMR** (126 MHz, Chloroform*-d*) δ 155.8, 147.3, 136.0, 135.0, 134.3, 129.4, 127.4, 126.1, 121.7, 40.2, 34.3, 32.5, 30.2, 27.1, 25.9, 19.6, 18.1, 17.4.

**1-(4-(*tert*-Butyl)phenyl)-3-(5-methyl-4-hydroxyhexyl)urea (9)**

To a solution of **8** (400 mg, 0.71 mmol) in tetrahydrofurane (10 mL), tetra-n-butylammonium fluoride (390 mg, 1.4 mmol) was added. The mixture was stirred at room temperature for 17 h. The crude was then diluted in ethyl acetate and washed with water twice and brine. The organic phase was dried over anhydrous magnesium sulphate. The solvent was evaporated, and the residue was purified by silica gel flash chromatography using a linear gradient of 0–10% methanol in dichloromethane as eluent. The compound **9** (200 mg, 0.65 mmol, 89%) was isolated as a white solid.

**MS-ESI(m/z)** calculated for C_18_H_31_N_2_O_2_ [M+H]^+^: 307.2; Found: 307.2.

**^1^H NMR** (500 MHz, Chloroform*-d*) δ 7.34–7.28 (m, 2H), 7.26–7.19 (m, 2H), 7.05 (s, 1H), 5.48 (t, *J =* 5.7 Hz, 1H), 3.35 (td, *J =* 5.5, 2.7 Hz, 1H), 3.31 (dd, *J =* 12.4, 5.9 Hz, 1H), 3.22 (dt, *J =* 12.9, 6.6 Hz, 1H), 1.73–1.51 (m, 3H), 1.50 (dtd, *J =* 12.4, 6.1, 3.1 Hz, 1H), 1.45–1.34 (m, 1H), 1.30 (s, 9H), 0.90 (dd, *J =* 6.8, 1.8 Hz, 6H).

**^13^C NMR** (126 MHz, Chloroform*-d*) δ 156.7, 146.6, 136.0, 126.0, 120.9, 40.1, 34.2, 33.7, 31.3, 30.9, 26.9, 18.8, 17.3.

**1-(4-(*tert*-Butyl)phenyl)-3-(5-methyl-4-oxohexyl)urea (10)**

To a solution of **9** (200 mg, 0.65 mmol) in dichloromethane (4 mL) was added silica (100 mg) and PCC (250 mg, 1.0 mmol). The suspension was stirred at room temperature for 4 h. More silica was added upon completion of the reaction followed by TLC then the solvent evaporated. The residue was purified by silica gel flash chromatography using a linear gradient of 0–50% ethyl acetate in cyclohexane as eluent. The product **10** (160 mg, 0.53 mmol, 71%) was obtained as a white solid.

**MS-ESI(m/z)** calculated for C_18_H_29_N_2_O*_2_* [M+H]^+^: 305.2; Found: 305.2.

**^1^H NMR** (500 MHz, Chloroform*-d*) δ 7.37–7.31 (m, 2H), 7.28–7.22 (m, 2H), 6.87 (s, 1H), 5.22 (t, *J =* 5.8 Hz, 1H), 3.24 (q, *J =* 6.6 Hz, 2H), 2.62 (p, *J =* 6.9 Hz, 1H), 2.55 (t, *J =* 6.8 Hz, 2H), 1.81 (q, *J =* 7.0 Hz, 2H), 1.32 (s, 9H), 1.10 (d, *J =* 6.9 Hz, 6H).

**^13^C NMR** (126 MHz, Chloroform*-d*) δ 215.2, 156.3, 146.8, 135.9, 126.1, 121.1, 40.9, 39.8, 37.5, 34.3, 31.3, 24.0, 18.3.

**9-((3a*S*,4*R*,6*S*,6a*R*)-6-Amino-2,2-dimethyltetrahydro-4H-cyclopenta[*d*][1,3]dioxol-4-yl)-9H-purin-6-amine (10)**

A solution of (1′*R*,2′*S*,3′*R*,4′*S*)-6-amino-9-[(4′-amino-2′,3′-*O*-isopropylidene)cyclopent-1′-yl]-9H-purine (155 mg, 0.55 mmol) and the **10** (420 mg, 1.4 mmol) and triethylamine (100 µL) in 5 mL methanol and stirred for 2 h. NaBH_3_CN (200 mg, 3.5 mmol) and AcOH (100 µL) were added. After 3 days, the solvent was evaporated and the residue was purified on silica gel flash chromatography using a linear gradient of 0–5% of methanol containing 0.1% ammonia in dichloromethane as eluent yielding two diastereoisomers

**11 Dia2** and **11-Dia1** were obtained with (0.15 mmol, 102 mg, 30%) and (0.16 mmol, 107 mg, 32%) yields, respectively, as white solids.

**1-((*R*)-4-(((3a*R*,4*S*,6*R*,6a*S*)-6-(6-amino-9*H*-purin-9-yl)-2,2-dimethyltetrahydro-4*H*-cyclopenta[*d*][1,3]dioxol-4-yl)amino)-5-methylhexyl)-3-(4-(tert-butyl)phenyl)urea (11-Dia1)**

**MS-ESI (m/z)** calculated for C_31_H_47_N_8_O_3_ [M+H]^+^: 579.3; found: 579.3.

**^1^H NMR** (500 MHz, DMSO*-d6*) δ 8.49 (s, 1H), 8.36 (s, 1H), 8.15 (s, 1H), 7.31–7.27 (m, 2H), 7.26–7.18 (m, 4H), 6.26 (t, *J* = 5.6 Hz, 1H), 5.00 (dd, *J* = 6.9, 4.4 Hz, 1H), 4.80 (td, *J* = 8.1, 4.3 Hz, 1H), 4.47 (dd, *J* = 7.0, 3.2 Hz, 1H), 4.11 (q, *J* = 5.3 Hz, 2H), 3.29 (d, *J* = 7.8 Hz, 1H), 3.18 (d, *J* = 5.0 Hz, 6H), 3.05 (q, *J* = 6.4 Hz, 2H), 2.33 (s, 1H), 2.18 (dt, *J* = 13.1, 8.2 Hz, 1H), 1.80–1.68 (m, 1H), 1.61 (s, 1H), 1.46 (s, 3H), 1.45–1.29 (m, 2H), 1.24 (s, 9H), 0.86 (dd, *J* = 24.3, 6.8 Hz, 6H).

**^13^C NMR** (126 MHz, DMSO*-d6*) δ 156.4, 155.8, 152.7, 149.8, 143.4, 140.5, 138.5, 125.6, 119.3, 117.8, 112.1, 85.8, 84.1, 70.2, 61.1, 60.3, 59.8, 49.0, 37.5, 34.2, 31.7, 30.2, 29.1, 28.0, 27.5, 27.2, 25.3, 19.2, 18.5.

**1-((*S*)-4-(((3a*R*,4*S*,6*R*,6a*S*)-6-(6-amino-9*H*-purin-9-yl)-2,2-dimethyltetrahydro-4*H*-cyclopenta[*d*][1,3]dioxol-4-yl)amino)-5-methylhexyl)-3-(4-(*tert*-butyl)phenyl)urea (11-Dia2)**

**^1^H NMR** (500 MHz, DMSO*-d6*) δ 8.42 (s, 1H), 8.32 (s, 1H), 8.14 (s, 1H), 7.33–7.26 (m, 2H), 7.22 (dd, *J* = 8.2, 6.1 Hz, 4H), 6.20 (t, *J* = 5.6 Hz, 1H), 5.02 (dd, *J* = 7.0, 4.7 Hz, 1H), 4.80 (ddd, *J* = 9.3, 7.3, 4.7 Hz, 1H), 4.51 (dd, *J* = 7.0, 3.4 Hz, 1H), 4.11 (d, *J* = 5.8 Hz, 1H), 3.32–3.25 (m, 1H), 3.18 (d, *J* = 3.0 Hz, 2H), 3.15–3.02 (m, *J* = 6.6 Hz, 2H), 2.50–2.43 (m, 1H), 2.36 (dd, *J* = 8.2, 4.0 Hz, 1H), 2.17 (dt, *J* = 12.9, 8.9 Hz, 1H), 1.85–1.74 (m, 1H), 1.63–1.55 (m, 1H), 1.46 (s, 3H), 1.38 (dt, *J* = 14.6, 7.1 Hz, 1H), 1.24 (d, *J* = 2.1 Hz, 9H), 0.85 (d, *J* = 6.9 Hz, 3H), 0.79 (d, *J* = 6.8 Hz, 3H).

**^13^C NMR** (126 MHz, DMSO*-d6*) δ 156.5, 155.8, 152.8, 149.7, 143.5, 140.5, 138.5, 125.6, 119.4, 117.8, 112.3, 85.8, 83.8, 60.7, 60.2, 59.7, 49.0, 37.9, 31.7, 31.6, 29.5, 27.6, 25.3, 19.2, 17.9.

To a solution of the **11-Dia1** (115 mg, 0.20 mmol) in 1mL of TFA was added 5µL of water and the mixture was stirred for 1 h, the volatile evaporated and the crude was purified on reverse phase AQ chromatography using a linear gradient of 0–100 acetonitrile in water as eluent, yielding **3-Dia1** (101 mg, 0.14 mmol, 98%) as a white powder

**1-((*R*)-4-(((1*S*,2*R*,3*S*,4*R*)-4-(6-amino-9*H*-purin-9-yl)-2,3-dihydroxycyclopentyl)amino)-5-methylhexyl)-3-(4-(*tert*-butyl)phenyl)urea (3-Dia1)**

**HRMS-ESI(m/z)** calculated for C_28_H_43_N_8_O_3_ [M+H]^+^: 539.3380; Found: 539.3460.

**^1^H NMR** (600 MHz, Deuterium Oxide) δ 8.35 (d, *J =* 13.7 Hz, 1H), 7.38–7.30 (m, 2H), 7.15–7.10 (m, 2H), 4.86 (dt, *J =* 10.9, 7.9 Hz, 1H), 4.59 (t, *J =* 7.4 Hz, 0H), 4.44 (td, *J =* 7.4, 6.7, 4.8 Hz, 1H), 3.83–3.76 (m, 1H), 3.36–3.30 (m, 1H), 3.31 (s, 1H), 3.21 (t, *J =* 6.4 Hz, 2H), 2.82 (dt, *J =* 13.1, 7.8 Hz, 1H), 2.68 (s, 1H), 2.44 (dt, *J =* 13.1, 10.5 Hz, 1H), 2.19 (pd, *J =* 7.0, 3.9 Hz, 1H), 1.83 (ddd, *J =* 14.6, 8.7, 5.5 Hz, 1H), 1.70 (ddd, *J =* 22.7, 11.6, 6.3 Hz, 1H), 1.66 (s, 2H), 1.23 (s, 8H), 1.22 (s, 1H), 1.02 (dd, *J =* 15.8, 6.9 Hz, 7H).

**^13^C NMR** (151 MHz, Deuterium Oxide) δ 158.3, 149.8, 148.5, 147.4, 144.1, 143.8, 135.4, 126.0, 120.9, 118.8, 73.3, 71.0, 63.3, 59.8, 59.3, 48.9, 38.9, 30.5, 28.7, 28.2, 26.1, 24.3, 16.9, 16.4.

To a solution of the **10-Dia2** (115 mg, 0.19 mmol) in 1mL of TFA was added 5 µL of water and the mixture was stirred for 1 h, the volatile evaporated and the crude purified on reverse phase AQ chromatography using a linear gradient of 0–100 acetonitrile in water as eluent, yielding **3-3 Dia2** (103 mg, 0.19 mmol, 96%) as a white powder

**1-((*S*)-4-(((1*S*,2*R*,3*S*,4*R*)-4-(6-amino-9H-purin-9-yl)-2,3-dihydroxycyclopentyl)amino)-5-methylhexyl)-3-(4-(*tert*-butyl)phenyl)urea (3-Dia-2)**

**HRMS-ESI(m/z)** calculated for C_28_H_43_N_8_O_3_ [M+H]^+^: 539.3380; Found: 539.3460

**^1^H NMR** (600 MHz, Deuterium Oxide) δ 8.35 (dd, *J =* 16.4, 1.6 Hz, 2H), 7.38–7.31 (m, 3H), 7.15–7.10 (m, 3H), 4.86 (q, *J =* 8.5 Hz, 1H), 4.59 (t, *J =* 7.6 Hz, 1H), 4.43 (s, 1H), 3.80 (td, *J =* 9.0, 4.8 Hz, 2H), 3.32 (dt, *J =* 6.3, 3.5 Hz, 2H), 3.21 (t, *J =* 6.5 Hz, 3H), 2.83 (dt, *J =* 14.3, 7.9 Hz, 2H), 2.68 (d, *J =* 1.6 Hz, 1H), 2.44 (q, *J =* 10.9 Hz, 2H), 2.19 (dd, *J =* 11.8, 6.7 Hz, 2H), 1.83 (dt, *J =* 14.8, 7.6 Hz, 2H), 1.72 (dt, *J =* 14.8, 7.0 Hz, 2H), 1.66 (p, *J =* 7.9 Hz, 4H), 1.23 (d, *J =* 1.6 Hz, 14H), 1.22 (s, 1H), 1.06–0.99 (m, 9H).

**^13^C NMR** (151 MHz, Deuterium Oxide) δ 158.4, 150.0, 148.5, 147.5, 144.4, 143.7, 135.4, 126.0, 121.0, 118.8, 73.4, 71.1, 63.3, 59.3, 59.3, 38.9, 30.6, 28.7, 28.2, 26.1, 24.3, 16.9, 16.4.

### 4.2. Biological Assays

#### DOT1L Enzymatic Inhibition Assays

The tests were carried out in 384-well white plates (Corning ref # CLS 3673). The recombinant DOT1L protein (1–416aa) is from Reaction Biology Corp (# HMT-11-101). Non-recombinant and unmethylated oligonucleosomes (Reaction biology Corp # HMT-35-130) containing 50 additional base pairs of internucleosomal DNA were purified from HeLa cells. The SAM (Sigma-Aldrich ref # A70007) was aliquoted in water at –80 °C. The reactions were performed in the “Assay Buffer” (AB) (50 mM Tris-HCl pH8, 150 mM NaCl, 3 mM MgCl_2_, 0.1% BSA). Protein, inhibitors, and oligonucleosomes were diluted in AB just before use. The poly-L-Lysine in the high salt buffer (50 mM Tris-HCl pH 7.4, 1M NaCl, 0.1% tween-20, 0.3% poly-L-Lysine) was purchased from Sigma Aldrich (ref # P1399) and stored in water at −20 °C. In a 384-well plate, the methylation reaction was initiated after a 10 min incubation of the compounds solutions (5 μL, 32 μM final) with the DOT1L enzyme solution (2.5 μL, 80 nM final). A solution containing the SAM cofactor and oligonucleosomes (2.5 μL, 2 μM, and 0.5 ng/well, respectively) was then added. After 45 min, the reaction was stopped by the addition of the high salt buffer containing poly-L-lysine and incubated for 15 min. A mix of anti-Histone H3 (C-ter) AlphaLISA acceptor beads (PerkinElmer, #AL147) (0.1 mg/mL final) and AlphaLISA biotinylated anti-dimethyl-Histone H3 Lysine 79 (H3K79) antibody (PerkinElmer, #AL148) (5 nM final) in detection buffer (DB) (AlphaLISA 5X Epigenetics Buffer 1 + AlphaLISA 30X Epigenetics Buffer Supplement, PerkinElmer, #AL008C1 & #AL008C2) were prepared and 5 µL of this mix were added for 1 h incubation at room temperature. For detection, 5 µL of Alpha Streptavidin Donor beads (PerkinElmer, #6760002) (0.1 mg/mL final) in DB were added and incubated for 30 min at room temperature. Finally, the plates were read using an EnVision 2103 multilabel plate reader (PerkinElmer) in AlphaLISA mode. Each point/concentration of the compounds was evaluated in triplicates per assay and the percentage of inhibition was calculated as the mean of at least three experiments. The percentage inhibition was calculated using the following equation:

(1) $$\%\;\mathrm{of}\;\mathrm{inhibition}=(1-(\frac{Xi-Xm}{XM-Xm}))*100,$$

where *Xi*, *XM*, *Xm* are the average signal at the considered concentration, minimal signal response (without enzyme and compound), and maximum signal response (without compound), respectively. Data analysis was performed using the GraphPad Prism 5 software. IC_50_ values were determined using the nonlinear regression fittings with sigmoidal dose-response (variable slope) function and the displayed EC_50_ are the mean of three independent experiments with associated standard deviations.

### 4.3. Cell Culture

Human leukemia cell line MV4-11 (CRL-9591) was purchased from American Type Culture Collection (Manassas, VA, USA). Cells were cultured in Roswell Park Memorial Institute (RPMI) 1640 Medium (RPMI) supplemented with 10% Fetal bovine serum (FBS) 5% GlutaMAX™ in order to prevent the accumulation of toxic ammonia and maintained in a humidified atmosphere at 37 °C and 5% CO_2_. Cells are reseeded every 4 to 5 days to 0.5 million cells/mL. All cell culture reagents were purchased from Gibco ThermoFisher Scientific.

The non-adherent cell lines MV4-11, MOLM-13, and KOPN8 exponentially growing cells were plated in 12-well plates (1 plate for each experiment) at a rate of 500,000 cells per well at a final volume of 2 mL. The cells were incubated in the presence of increasing inhibitor concentrations (0.1, 0.32, 1, 3.2, 10 µM). The cells (1 to 2 million) were harvested at the time chosen according to the line in order to extract the histones and the RNAs.

For assessment of cell proliferation and viability in human cell lines, exponentially growing cells were placed, in triplicate, in 96-well plates at a density of 35,000 cells/well at a final volume of 100 µL. Cells were incubated in the presence of 1, 3.2, and 10 µM of **3 Dia2** and of EPZ-5676, respectively. The viable cell number was determined every 3–4 days for up to 15 days to 21 days using trypan blue counting using Cellometer Disposable Counting Chambers for the Cellometer Mini Automated Cell Counter.

### 4.4. Histone Extraction

For the isolation of histones from cells, 1 to 2 million cells were collected, after exposure to the chosen treatment, by centrifugation at 200× *g* and lysed by 5 min of incubation on ice in 250 μL of the nuclear extraction buffer (10 mM Tris-HCl, 10 mM MgCl_2_, 25 mM KCl, 1% Triton X-100, 8.6% sucrose, Roche Protease inhibitor tablet 1836145). The nuclei were collected by centrifugation at 600× *g* for 5 min at 4 °C. The supernatant was removed and the histones were extracted for 1 h with 0.4 N cold sulfuric acid. The extracts were clarified by centrifugation at 10,000× *g* for 10 min at 4 °C and transferred to a microcentrifuge tube containing a 10× volume of ice-cold acetone. The histones were precipitated at ‒20 °C overnight and recovered by centrifugation at 1500× *g* for 10 min in the form of a pellet and then resuspended in 80 μL of water. Histones were quantified by absorbance measured by a nanodrop at 276 nm.

### 4.5. Western Blotting

After extraction, the histones (500ng) were separated on 4–20% Tris-Glycine gels (Invitrogen) and transferred onto a nitrocellulose membrane (0.2 mm) using the iBlot^®^ Gel Transfer Stacks Nitrocellulose kit Mini (Invitrogen). After blocking nonspecific sites for 1 h at room temperature in a solution of 1X TBS at 5% milk, the membrane was incubated with rabbit anti-H3K79me2 (ab3594, 1/1000) or anti-H3 (ab1791)1/1000) overnight at 4 °C or 1 h at RT. The membrane was then washed three times for 5 min in a 1X TBS solution containing 0.05% Tween20 and was then incubated with an HRP anti-rabbit secondary antibody (NA934V, 1/10,000) for 1 h at RT. After 3 washes of the secondary antibody, the membrane was revealed using the ECL™ Prime Western Blotting Detection Reagents Developer Kit (GE Healthcare). The PageRuler prestained protein ladder 10 to 180 kDa (Fisher Scientific) was used as a molecular weight marker. The antibody-labeling of histone H3 was used as an internal control for loading and amount of histones in each well. At least three independent experiments were run.

### 4.6. Flow Cytometric Analysis of Cell Cycle and Apoptosis

In a 12-well plate, 2 mL exponentially growing MV4-11 cells were plated with a density of 2 × 10^5^ cells/mL. Cells were incubated at a final concentration of 1 µM or 3.2 µM 3 Dia2 and 1 µM EPZ-5676 for 12 days. Culture media and drugs were replaced every 2–3 days and cells were split back to a density of 2 × 10^5^ cells/mL at the same time. Cells were harvested on days 8, 10, 12 and split to allow the analysis of cell cycle and Annexin V staining simultaneously. The apoptosis ratio was detected by an Annexin V-APC/7-AAD double staining assay (SONY 3804610) and cells were prepared according to the manufacturer’s recommendations. Cells for the cell cycle analysis were collected by centrifugation at 500× *g* for 5 min at 4 °C. The pellets were washed once with cold PBS and then fixed with 70% ethanol on ice for 30 min. Afterwards, the cells were washed once with cold PBS and stained with propidium iodide (Invitrogen P3566) for 30 min at 37 °C. Samples were measured using an Attune NxT acoustic focusing cytometer (Life Technologies), data were analyzed by FlowJo.

### 4.7. Quantitative PCR Real Time

Total RNA was isolated from the cell pellet of at least 1 × 10^6^ cells using the RNAeasy mini kit (Cat No.: 74106, Qiagen) according to the manufacturer’s instructions. Approximately 500 ng of total RNA was reverse transcribed using PrimeScript™ RT Master Mix (Perfect Real Time) (Cat No.: RR036B, Takara Bio Europe, Saint Germain-en-Laye) according to the manufacturer’s instructions. Complementary DNA (cDNA) was diluted at 1:10 and used as a template in quantitative real-time PCR (RT-qPCR) using the SYBR Green Master Kit (ref. 047516001, Roche, Boulogne-Billancourt, France) and LightCycler^®^480 (Roche, Boulogne-Billancourt, France) System according to the manufacturer’s instructions. The cycling conditions were denaturation at 95 °C for 5 min, followed by 40 cycles of amplification at 95 °C for 10 s, one cycle of hybridization at 49 °C for 20 s, and one cycle of elongation at 72 °C for 16 s.

Target gene (HOXA9 and MEIS1) cycle numbers were normalized to the housekeeping gene TATA-binding protein (TBP), β2-microglobulin (B2M) or Tyrosine 3-Monooxygenase/Tryptophan 5-Monooxygenase Activation Protein Zeta (YWHAZ) to obtain a ΔCT value normalized to vehicle control (cells treated with 0.1% DMSO). The fold change in relative mRNA expression was calculated using the Livak method and the equation (2^‒ΔΔCT^), where the ΔΔCT is the difference between the normalized target gene and vehicle control (ΔCT sample ‒ ΔCT control = ΔΔCT) (Table 2).

## Figures and Tables

**Table 1:** The mean value of EC_50_ (nM) of the proliferation inhibition for selected MLLr cell lines after 15-day treatment (mean value of two independent experiments run in triplicate and standard error).

Cell Line	Dia2	EPZ-5676	Doubling Time
MV4-11 (AF4)	93 ± 4	21 ± 1	12 h
KOPN8 (ENL)	63 ± 2	86 ± 4	48 h
NOMO-1 (AF9)	660 ± 20	562 ± 15	30 h
MOLM-13 (AF9)	120 ± 10	92 ± 2	22 h

**Table 2:** Sequence of the qPCR primers.

HOXA9-F2	CCCCCATCGATCCCAATAACCC
HOXA9-R2	CCAGTTCCAGGGTCTGGTGTT
MEIS1-F1	CGATGGCGCAAAGGTACGAC
MEIS-1-R2	ATGCGGGTCCCCATACATCG
B2M-F	GAGATGTCTCGCTCCGTGGC
B2M-R	CCTGAATCTTTGGAGTACGCTGG
TBP	TTACCGCAGCAAACCGCTTG
TBP	TTGACCTAAAGACCATTGCACTCCGT
YWHAZ (U/L)	CCCTCAAACCTTGCTTCTAGGAGA
YWHAZ (U/L)	TCATATCGCTCAGCCTGCTCG

## Data Availability

Complementary data are available in the Supplementary Information.

## Supplementary Materials

The following are available online, Figure S1: Presentation of co-crystal ligand EPZ004777 (magenta) and best three re docked ligands in the active pocket ofDOT1L (left) and important amino acid residues included in interaction with co-crystal ligand (right), Figure S2: Western blot used for Figure 7, Figure S3: Cell cycle analysis of MV4-11 cells treated with 3-Dia-2 over time. Inhibition of cell cycle progress on MV4-11cells treated with compound Dia-2 at 1 μM or 3.2 μM and EPZ- 5676 at 1 μM. Cells treated with an equal amount of DMSOwere used as negative controls. Cells were fixed with ethanol and stained with propidium iodide. Cell cycle distributionwas analyzed by a flow cytometry. One representative experiment is reported and its quantification, Figure S4: Cell apoptosis analysis of MV4-11 cells treated with 3-Dia-2 over time. Apoptosis ratio detection by AnnexinV-APC/7-AAD double staining assay, analyzed by a flow cytometry on MV4-11 cells treated with compound 3-Dia-2 at 1 μM or 3.2 μM and EPZ-5676 at 1 μM. The Q1 area represents damaged cells appearing in the process of cell collection, theQ2 area represents necrotic/late period apoptotic cells, the Q3 area represents early apoptotic cells, and the Q4 area representsthe normal cells. One representative experiment is reported and its quantification, Figure S4: Selectivity evaluation of the compound **Dia-2** by fluorescence microscopy HCS. (**A**) fluorescence imaging of theH3K79me2 mark (**B**) bar diagram of the percentage of inhibition for compound **Dia-2**, Table S1: Selectivity of the compound on a HMT panel (by Reaction Biology, USA) and on hDNMT3Acat (as described in [39]).

## Sample Availability

Samples of the compounds Dia-1 and Dia-2 are available from the authors.

## Abbreviations

DIAD	Diisopropyl azodicarboxylate
DOTIL	Disruptor of telomeric silencing 1-like H3K79: 79th lysine of the histone 3 MLL: Mixed Lineage Leukemia
MLLr	Mixed-lineage rearranged leukemia
PCC	Pyridinium chlorochromate
SAM	*S*-Adenosyl-L-methionine
TBAF	Tetrabutylammonium fluoride
TBDPSCl	tert-Butyldiphenylchlorosilane
