# Supplementary material for: Synthesis and Biological Activity of a Cytostatic Inhibitor of MLLr Leukemia Targeting the DOT1L Protein
Source: Molecules. 2021 Aug 31;26(17):5300. doi: 10.3390/molecules26175300 (PMC8434109; doi:10.3390/molecules26175300)

Supplementary Materials

Synthesis and Biological Activity of a Cytostatic Inhibitor of MLLr Leukemia Targeting the DOT1L Protein

Corentin Bon [1,2], Yang Si [1], Melanie Pernak [1], Magadalena Barbachowska [1,2], Veronique Cadet Daniel [1], Dusan Ruzic [3], Nemanja Djokovic [3], Teodora Đikić [3], Katarina Nikolic [3], Ludovic Halby [1] and Paola B. Arimondo [1,*]

[1] Epigenetic Chemical Biology, Department of Structural Biology and Chemistry, Institut Pasteur, UMR3523 CNRS, 75015 Paris, France, corentin.bon@pasteur.fr (C.B.); sophia.siyang@gmail.com (Y.S.); m.pernak@sfr.fr (M.P.); magdalena.barbachowska@pasteur.fr (M.B.); veronique.cadet-daniel@pasteur.fr (V.C.D.); ludovic.halby@pasteur.fr (L.H.)

[2] Ecole Doctorale MTCI, Université de Paris, Sorbonne Paris Cité, Paris, France, magdalena.barbachowska@pasteur.fr

[3] Department of Pharmaceutical Chemistry, Faculty of Pharmacy, University of Belgrade, Vojvode Stepe 450, 11000 Belgrade, Serbia, dusan.ruzic@pharmacy.bg.ac.rs (D.R.); nemanja.djokovic@pharmacy.bg.ac.rs (N.D.); teodora.djikic@pharmacy.bg.ac.rs (T.D.); katarina.nikolic@pharmacy.bg.ac.rs (K.N.)

* Correspondence: paola.arimondo@cnrs.fr1

Molecular docking

To examine the binding modes and rank the studied compounds **Dia 1** and **Dia 2** according to their affinity to DOT1L, we performed molecular docking study in GOLD 5.8.1 Software (https://www.ccdc.cam.ac.uk/solutions/csd-discovery/Components/Gold/). The crystal structure of DOT1L enzyme (PDB: 4ER3) was used for molecular docking study, whereas Discovery studio Visualizer v16.1.0 was used for visualization of the interactions between hit molecules and DOT1L.

To prepare valid setup for correct prediction of pose and orientation of studied inhibitors, we first prepared different settings and investigated which one has satisfactory reproducibility. The parameter which is used for docking validation is RMSD (root mean square deviation of atomic position). It is described in the literature that valid molecular docking setup is achieved when RMSD value is < 2Ǻ [doi.org/10.1021/ci800293n]. The binding pocket was defined according to the atomic coordinates of co-crystal ligand (all the atoms around co-crystal ligand in diameter of 6Ǻ were considered for potential interactions). We enabled for co-crystal ligand and synthesized compounds to rotate around flexible bonds. After several settings, we concluded that synthesized derivatives with the bulky para-tert-butylphenyl group were not able to anchor in the hydrophobic part of cofactor's pocket. Therefore, we decided to make some of the closest hydrophobic amino acid residues flexible. The residue we found that has the most prominent effect on the anchorage of para-tert-butylphenyl moiety is Phe245. This phenylalanine behaves like a gatekeeper in the hydrophobic cleft, enabling bulkier groups to reach the hydrophobic subpocket. The final calculations were based on the Gold Score as a scoring function.

The co-crystal ligand (EPZ004777) from crystal structure of DOT1L was re-docked in the active pocket of examined enzyme and RMSD value was calculated. GOLD software calculated this value equal to 0.6117 Ǻ, which is considered as valid docking setup for further testing of ligands. The presentation of co-crystal and re-docked structure of EPZ004777 is shown on **Figure S5**. From these results we concluded that re-docked ligand made the same interactions with the amino acid residues in the cofactor pocket of DOT1L as co-crystal ligand.

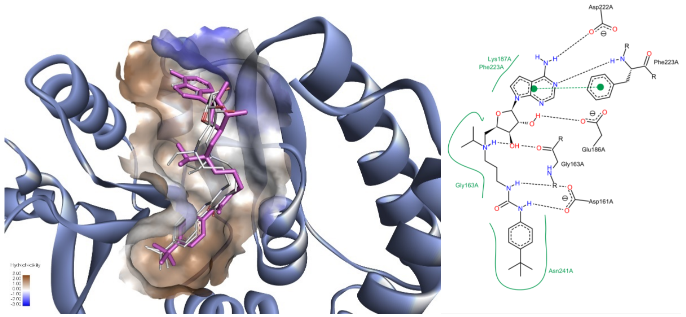

Figure S1: Presentation of co-crystal ligand EPZ004777 (magenta) and best three re docked ligands in the active pocket of DOT1L (left) and important amino acid residues included in interaction with co-crystal ligand (right).

Supplementary Figures

Figure S2. Western blot used for Figure 7.

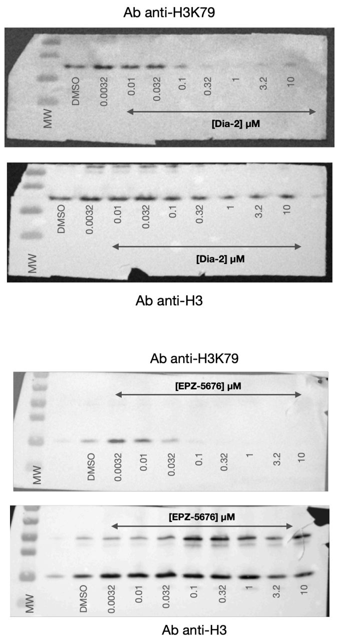

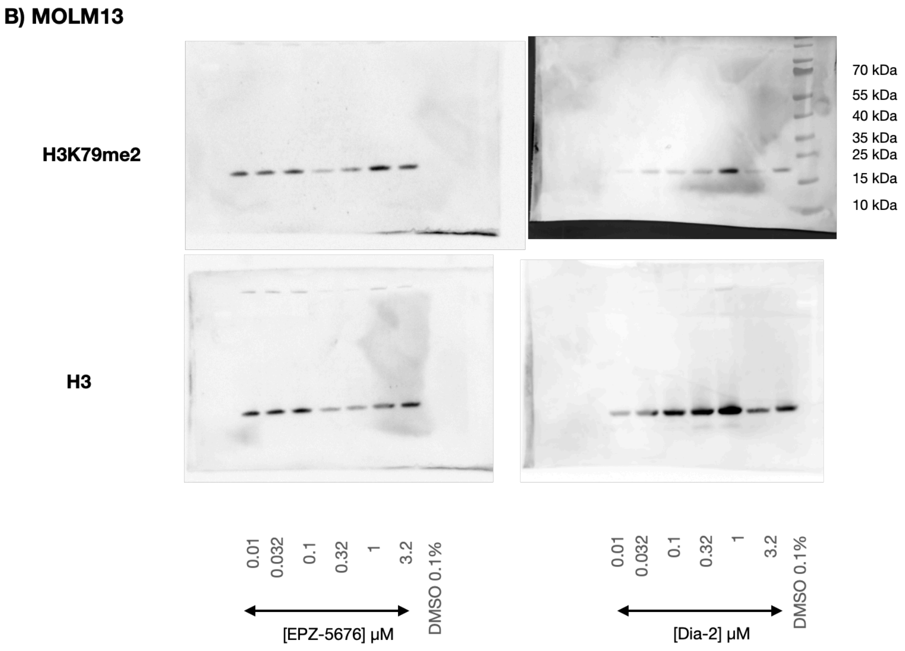

Figure S3. Cell cycle analysis of MV4-11 cells treated with 3-Dia-2 over time. Inhibition of cell cycle progress on MV4-11 cells treated with compound Dia-2 at 1 μM or 3.2 μM and EPZ- 5676 at 1 μM. Cells treated with an equal amount of DMSO were used as negative controls. Cells were fixed with ethanol and stained with propidium iodide. Cell cycle distribution was analyzed by a flow cytometry. One representative experiment is reported and its quantification.

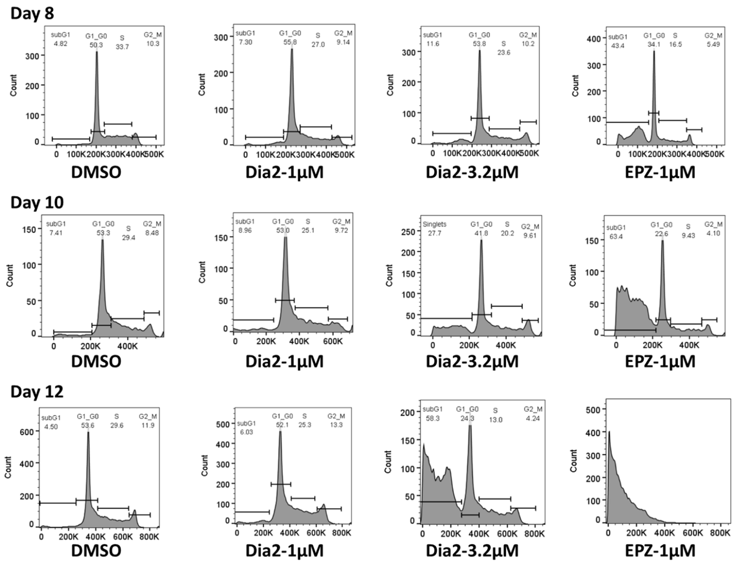

Figure S4. Cell apoptosis analysis of MV4-11 cells treated with 3-Dia-2 over time. Apoptosis ratio detection by Annexin V-APC/7-AAD double staining assay, analyzed by a flow cytometry on MV4-11 cells treated with compound 3-Dia-2 at 1 μM or 3.2 μM and EPZ-5676 at 1 μM. The Q1 area represents damaged cells appearing in the process of cell collection, the Q2 area represents necrotic/late period apoptotic cells, the Q3 area represents early apoptotic cells, and the Q4 area represents the normal cells. One representative experiment is reported and its quantification.

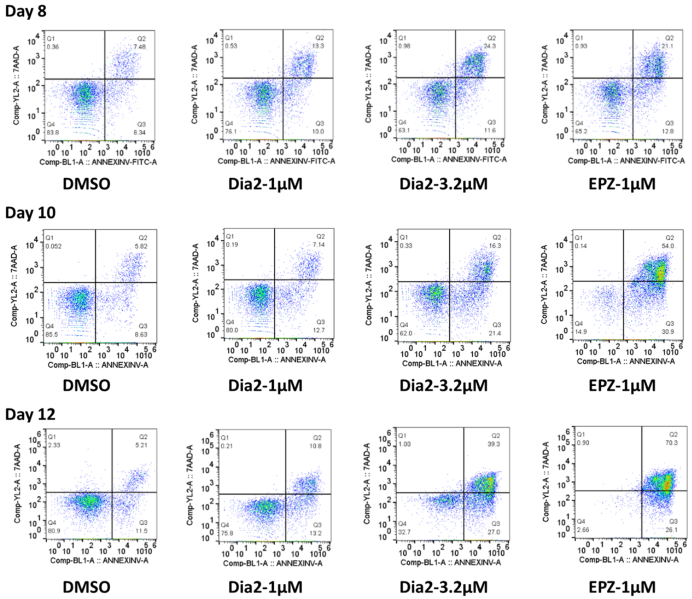

Table S1: Selectivity of the compound on a HMT panel (by Reaction Biology, USA) and on hDNMT3Acat (as described in Ceccaldi *et al.* ChemBioChem 2011, Volume 12, Issue 9 p. 1337-1345).

		Enzyme % Activity (relative to DMSO controls)
Compound ID		3-Dia2
Testing Concentration		10µM
Methyltransferase:	Substrate:	Activity enzyme in %
EZH2 Complex	Core Histone	86±1
G9a	Histone H3 1-21	97±1
PRMT4	Histone H3	92±1
PRMT6	GST-GAR	95±1
SET7/9	Core Histone	82±1
SMYD2	Histone H4	92±1
DNMT3A	DNA	96±2

Figure S4. Selectivity evaluation of the compound **Dia-2** by fluorescence microscopy HCS. A) fluorescence imaging of the H3K79me2 mark B) bar diagram of the percentage of inhibition for compound **Dia-2.**

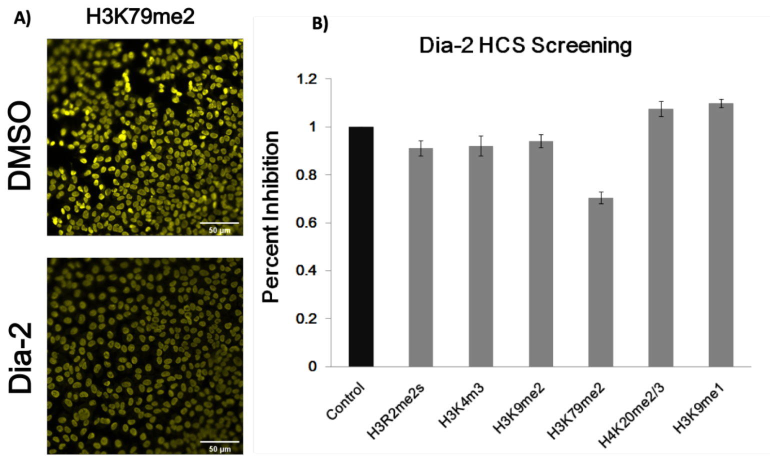

Supplement: Supplementary file 1 [file molecules-26-05300-s001.zip › molecules-1321863-supplementary.pdf]
